# Combining a parsimonious mathematical model with infection data from tailor-made experiments to understand environmental transmission

**DOI:** 10.1038/s41598-023-38817-z

**Published:** 2023-08-10

**Authors:** Anna M. Gamża, Thomas J. Hagenaars, Miriam G. J. Koene, Mart C. M. de Jong

**Affiliations:** 1 Quantitative Veterinary Epidemiology, Wageningen University and Research, 6708 PB Wageningen, The Netherlands; 2 Wageningen Bioveterinary Research, Wageningen University and Research, 8221 RA Lelystad, The Netherlands

**Keywords:** Infectious diseases, Bacterial infection, Bacteriology, Pathogens, Computational models, Data processing

## Abstract

Although most infections are transmitted through the environment, the processes underlying the environmental stage of transmission are still poorly understood for most systems. Improved understanding of the environmental transmission dynamics is important for effective non-pharmaceutical intervention strategies. To study the mechanisms underlying environmental transmission we formulated a parsimonious modelling framework including hypothesised mechanisms of pathogen dispersion and decay. To calibrate and validate the model, we conducted a series of experiments studying distance-dependent transmission of *Campylobacter jejuni* in broilers. We obtained informative simultaneous estimates for all three model parameters: the parameter of *C. jejuni* inactivation, the diffusion coefficient describing pathogen dispersion, and the transmission rate parameter. The time and distance dependence of transmission in the fitted model is quantitatively consistent with marked spatiotemporal patterns in the experimental observations. These results, for *C. jejuni* in broilers, show that the application of our modelling framework to suitable transmission data can provide mechanistic insight in environmental pathogen transmission.

## Introduction

Traditionally, transmission of infectious diseases is modelled as a process that occurs when a susceptible host has direct contact with an infectious host^1^. However, the majority of pathogens is in fact transmitted through the environment, i.e. indirectly: through the air, via surfaces and/or via fomites whilst residing in droplets, dust particles or otherwise. For pathogens that spread in this fashion, infectious material is shed into the environment by the infectious host (the source) and it is taken up (e.g. ingested, inhaled, or absorbed through mucosa) by a susceptible recipient host after the material has spent any amount of time in the environment. During this time, infectious material is losing infectivity due to inactivation (decay) of pathogens as these are exposed to environmental conditions that often are not optimal for their survival^2^. Additionally, part of emitted infectious particles may never produce any exposure to a recipient host, for example by being deposited in locations inaccessible to the hosts. Furthermore, data from indirect transmission experiments confirm that infectious material can also be dispersed through the environment, for example being moved via air flow, water movement, or movements of contaminated objects i.e. fomites^3–8^. This may cause the material to be moved from areas that are close to the source to parts of the environment more distant from the source, thereby reducing the probability of infection near the source and at the same time facilitating uptake by distant recipient hosts. Depending on the speed of dispersion, material arriving at larger distances from the source is expected to have undergone more inactivation due to a longer travelling time. Thus, the processes of shedding, decay and dispersal interact to shape the overall spatiotemporal rate of pathogen transmission through the environment.

The environmental stage of transmission provides opportunities for non-pharmaceutical interventions aimed at reducing transmission. Some of these, such as separation of hosts (“social distancing”) and hygiene protocols, are applied to the infectious and/or recipient individuals; while others, such as disinfection procedures or ventilation measures, are applied to the environment occupied by the hosts. To develop the best (combination of) intervention strategies and quantify their efficacy, mechanistic mathematical models of transmission are needed that are both calibrated (i.e. having identifiable parameters) and validated.

Here we present an experimentally validated modelling framework to mechanistically model environment dependent processes, namely pathogen decay and dispersion. We use a parsimonious modelling approach, motivated by the fact that detailed transmission mechanisms in host–pathogen-environment systems are generally difficult to observe and measure. Parsimonious models can be used even when limited observations are available. In addition, such models can complement and validate more detailed modelling of very specific hypothesised transmission routes, e.g. based on aerosol physics^9,10^.

Mechanistic models of environmental transmission that not only account for pathogen survival but also for movement of infectious material are still under development. As it is described in Lanzas et al.^11^ two main types of mechanistic models are commonly used to describe environmental transmission: “mean field” compartmental models, versus individual based models. Mean field models, that describe the environment as a compartment (reservoir) that contains infectious material deposited by infectious hosts (described as a compartment) and accessed by recipient hosts (also described as a compartment) were developed for bacterial (e.g. cholera^12,13^), viral (e.g. avian influenza^14^ or hepatitis E in pigs^15^), prion (e.g. chronic wasting disease^16^) and parasitic (e.g. *Cryptosporidium*^17^) infections. Individual based models, that describe the hosts (and sometimes also pathogens) not as a group but as a set of individuals that interact with each other and that share a spatially explicit environment, were also developed for many systems, including bacterial (e.g. *E.coli*^18^ or *Clostridium difficile*^19^), viral (e.g. influenza^20^) and parasitic (e.g. *Taenia solium*^21^ or *Toxoplasma gondii*^22^) infections.

Both the mean-field and spatially explicit models usually do not account for movement of infectious material through the environment.

The simplest mechanistic description for environmental dispersion of material, that is in our case carrying a certain pathogen load, is as a diffusion process^23^. Up until now in epidemiological models, diffusion, or reaction–diffusion, has been mainly used for describing diffusion of hosts, as for example in Huang et al.^24^. For environmental transmission, diffusion models were also developed, few of which implemented diffusion of pathogens or infectious material for plant^25–27^ and animal or human diseases^28–31^. While the vast majority of transmission models accounting for dispersion of infectious material remains theoretical, for some more complex, simulation models a validation to observations was reported (for airborne^32,33^ and waterborne^34^ transmission).

In a more general context, the importance of model calibration and validation for zoonotic environmentally transmitted infections, such as *Campylobacter spp.*, was recently raised in Rees et al.^35^; the authors noted that less than half of the 210 analysed models were validated with real-life data emphasizing the need for modelling that is driven by actual transmission data.

To calibrate and validate an epidemiological model two types of transmission data can be used: field data and data from controlled transmission experiments. The most common information source is field data collected by detecting naturally occurring infections in the areas where the pathogen is prevalent or emerging. As field data represent naturally occurring transmission chains, often there is little control over the quantity and quality of the data. Moreover, linking infection events to infectious source individuals is challenging as the contact structure and chain of infections cannot always be observed and/or verified.

Alternatively, the data can be collected from tailor-made transmission experiments conducted using animal models^36^. In transmission experiments the net effect of the entire process, i.e. the shedding by infectious hosts, the environmental stage of the pathogen and the exposure response of recipient hosts, can be studied in controlled (environmental) conditions. Both source and recipient hosts can easily be identified by starting the experiments in a clean (pathogen free) environment and recording the status of all hosts before and during the observation period. The experiments can be tailored to the spatiotemporal resolution necessary for the system of interest, so that a mathematical model at hand can be calibrated and validated. The last property is especially important for systems where identifiability problems cannot be solved by additional data on specific mechanisms.

An environmental transmission model accounting for pathogen decay and diffusion was presented previously in van Bunnik et al.^3^. The four-parameter model was fitted with data obtained in experiments on *Campylobacter jejuni* (in the remainder of this paper abbreviated as *C. jejuni*) and *Escherichia coli* transmission between broilers spatially distanced by a single distance band (between 0.75 and 1.06 m) combined with a separate survival experiment in which concentration of culturable bacteria in faeces was measured. This separate survival experiment was needed to solve identifiability problems in the model; here we prove that also our three-parameter model describing decay and diffusion of infectious material is not identifiable with the previously published data on *C. jejuni* transmission. To show that the model can be fully identifiable from transmission data only when sufficient spatiotemporal resolution is provided, we conducted new experiments for the same system, varying the distance and the timing of exposure. With the addition of the spatiotemporal data from these new experiments we were able to estimate all parameters, i.e. also the decay rate parameter, with remarkable results when compared to the estimate obtained from the separate survival experiment. Subsequently, we quantitatively validated both time and distance dependence of the model showing that the model fit is consistent with distance dependent delay times and proportions of hosts colonised observed in experiments.

Our approach enabled us to simultaneously study pathogen decay and dispersion in the environment using parsimonious modelling and spatiotemporal data from transmission experiments only and hence obtain new insights into mechanisms underlying environmental transmission of *C. jejuni*. One of the insights being that separate experiments counting culturable bacteria in the environment may not provide information representative for the decay rate associated with the infectious environmental stages of the *C. jejuni*.

## Results

### Model

To study environmental transmission we developed a spatiotemporal three-parameter model, where each parameter has a precise biological interpretation. A decay rate parameter α describes how fast *C. jejuni* is inactivated in the environment. A diffusion coefficient D describes how the spatial distribution of infectious material in the environment changes over time, as a result of movement/dispersal of this material. A transmission rate parameter β describes the hazard rate of infection for one unit of exposure. It reflects the joint effects of the remaining host-dependent processes, namely strength of shedding of infectious material by source hosts, and the exposure response (i.e. infection or colonisation) of the recipient. The unit of exposure is here defined as the hypothetical exposure of a recipient to the total infectious material produced by one infectious individual during one time unit of shedding and over one unit of source area.

For a standard compartmental model extended with an environmental reservoir with spatially homogeneous infection load, the probability of infection can be represented as:

1 $${P}_{\text{inf}}\left({t}_{1}\text{,}{t}_{2}\right)=1-\mathrm{exp}\left[-\beta \frac{\overline{W }\times \left({t}_{2}-{t}_{1}\right)}{N}\right],$$

where β is a transmission rate parameter, N the number of hosts, and $\overline{W }$ is the average environmental exposure during a time interval from t_1_ to t_2_:

$$\overline{W }=\frac{{\int }_{{t}_{1}}^{{t}_{2}}W\left(t\right)dt}{\left({t}_{2}-{t}_{1}\right)}.$$

In our spatial model, the probability of infection is given by a spatially non-homogeneous generalization of Eq. 1. It represents the exposure response for a given recipient host occupying an exposure area A_exp_ during a time interval t_1_ to t_2_ and reads as follows:

2 $${\mathrm{P}}_{\text{inf}}\left({t}_{1}\text{,}{t}_{2}\text{,} \, {A}_{\text{exp}}\right)=1-\mathrm{exp}[-\beta {\int }_{{t}_{1}}^{{t}_{2}}{\iint }_{{A}_{\text{exp}}}W\left(t,x, y\right) dx \, dy \, dt ],$$

where β (again) is a transmission rate parameter and W(t,x,y) is the spatiotemporal density function of the environmental load the recipient is exposed to. W describes how the distribution of the accumulated load changes in time and space, and in our model accounts for continuous shedding, exponential decay and diffusion of material. For further details on the modelling we refer to the “Methods” section.

### Experimental results

To calibrate and validate the model we conducted a series of animal experiments studying transmission of *C. jejuni* between spatially separated broilers. Data on infection were gathered by recording colonization status of the recipient hosts at various locations and at various time points. In earlier transmission experiments on *C. jejuni* broilers were housed in pens separated from the source by a single distance band (between 0.75 and 1.06 m)^3^ and this narrow distance range proved to be insufficient for estimation of all of the model parameters nor validation of the distance dependence of transmission. In the current study, we designed experiments to compare transmission in pens that were separated by a much broader distance range (0.00–2.00 m). In all those experiments, five broilers were inoculated with *C. jejuni* and placed in an experimental room at day 0. Two types of experiments with slightly different design were conducted: type 1 experiments where exposure of recipients started the same day as the source animals were inoculated and type 2 experiment where the exposure started 20 day after inoculation of the source broilers. This allowed us to validate whether the delay in (onset of) transmission across a distance, as reported in van Bunnik et al.^3^, is shorter or not, when transmission starts in a contaminated environment, as predicted by our spatiotemporal model. Table 1 sums up the most important differences between the two types of experiments.

**Table 1:** Summary of experimental design for *C. jejuni* transmission experiments between separated broilers.

		Experiment type 1	Experiment type 2
Duration	[day]	35	35
Time of inoculation of source hosts	[day]	0	0
Start of exposure of recipient hosts	[day]	0	20
Border to border distance ranges between source and recipient host areas	[m]	0.35–2.00	0.43–0.89^1^ and 0.00^2^
Number of hosts per recipient pen		1	2
Recipients removed after found positive		Yes	No
Experimental groups		NA	A, B, C

^1^Group A.

^2^Groups B and C.

In the type 2 experiment, to gather data on transmission by direct contact, i.e. on extremely short distances (0.00 m), beside distanced recipients (group A) we included two additional pairwise groups. Group B consisted of recipients housed together with inoculated animals (source hosts) also from day 20 onwards. Group C consisted of recipients housed with another host that initially was a recipient host and turned into a source host by becoming infected. We note that in group C the recipients were not only exposed to the infectious material of their colonized pen mate but also to material originating from the distanced sources; however our modelling indicates that the contribution of the latter sources to the total exposure of a group-C recipient is relatively small (see Supplementary Note S4 for details).

**Figure 1:**
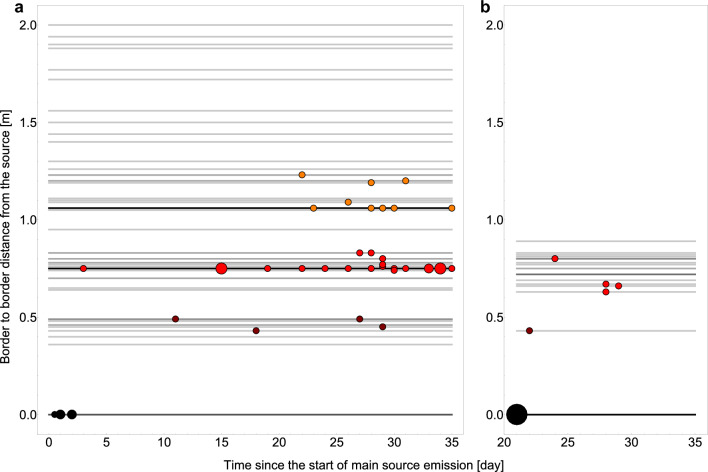
Results from experiments on transmission of *C. jejuni* between spatially separated broilers; (**a**) results from all type 1 experiments where exposure started the same day as source broilers (main source) were inoculated and group C from type 2 experiment that were housed with their pen-mate broiler (main source) the day that pen mate started shedding *C. jejuni*; (**b**) results from recipients from type 2 experiment where exposure started 20 days after inoculation of source broilers (main source) for group A (housed in pens distanced from the main source) and B (housed in the same pens as main source broilers); to present the experimental outcome in a clear and intuitive way we simplified and aggregated the data; detailed experimental outcome is provided in supplementary materials S1; point size is scaled with the case number for that particular time and distance, and colour indicates one of 4 distance bins (black: 0 m, dark red: 0.35–0.60 m, red: 0.61–1.00 m, orange: 1.01–1.30 m); grey lines mark the distances of all individual pens used in experiments; pens that housed both source and recipient hosts are shown as distanced by 0 m; line colour is scaled with number of pens for that particular distance (darker for more pens).

Figure 1 shows the outcome of experiments as a function of time from the start of experiment (start of the exposure) and as a function of border to border distance between source area and recipient area. Source areas are defined as pens occupied by colonized hosts, while recipient areas are defined as pens occupied by recipient hosts (non-colonised when exposure started). When an area contained both colonized and non-colonized broilers it therefore was both a source and a recipient area.

For non-zero distances between source and recipient, the experiments displayed a time delay between start of recipient exposure until onset of recipient infections, and this delay time increased with distance. As expected, the delay observed when recipient hosts were placed in an already contaminated environment (type 2 experiment, group A) was shorter than the delay observed for the same distance range when the environment was not contaminated prior to the start of exposure (type 1 experiments). For the pairwise experimental groups (group B and C), where we studied transmission between broilers that were housed together (separated by 0 m distance) very fast onset of transmission was observed (as expected). In agreement with the prediction of our spatiotemporal model mentioned above, for group B, that was exposed to an environment previously contaminated by their pen-mates (being the main source), transmission was faster than for group C, where there was no previous contamination, by the infected pen mate (being the main source), as exposure to the infected pen mate as main source started on the same day that this pen mate started shedding.

### Parameter estimates

First, we estimated all three parameters (by maximum-likelihood estimation) through a model fit to the data from three previously published type 1 experiments where only a single distance band was studied^3,8^. The estimated decay rate parameter α was 0.000 day^−1^ (CI 0.000–0.083), the transmission rate parameter β was 0.008 day^−1^ (CI 0.005–0.027), and the diffusion coefficient D was 0.089 m2 day−1 (CI 0.026–0.826). The profile likelihood plots are provided in Supplementary Note S2. The decay rate parameter α estimated to be 0 day^−1^ means that the decay time is estimated to be indefinitely long, i.e. the parameter is practically unidentifiable. Therefore, we conclude that the three-parameter model is non-identifiable with the (previously published) data of only a single distance band. Also, the value α = 0 day^−1^ is clearly nonbiological as even for pathogens with long survival in the environment a finite survival time is expected.

**Figure 2:**
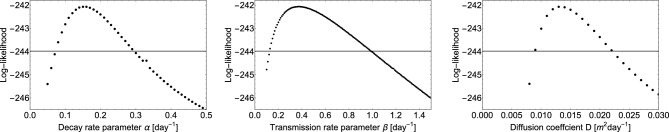
Profile likelihoods for model parameters: decay rate parameter α, transmission rate parameter β and diffusion coefficient D, horizontal lines mark the likelihood value for the confidence bounds.

Next, we estimated all three parameters through a model fit to the joint data of the transmission experiments, thus additionally including the new experimental data (one type 1 and one type 2 experiment) studying transmission for varying distance bands. The estimated decay rate parameter α was 0.153 day^−1^ (CI 0.072–0.295), the transmission rate parameter β was 0.372 day^−1^ (CI 0.125–0.989), and the diffusion coefficient D was 0.013 m^2^ day^−1^ (CI 0.008–0.023). Univariate profile likelihoods for all three parameters are shown in Fig. 2. Finite confidence intervals estimated for parameters show that all three parameters were identifiable.

### Fit to experimental data

**Figure 3:**
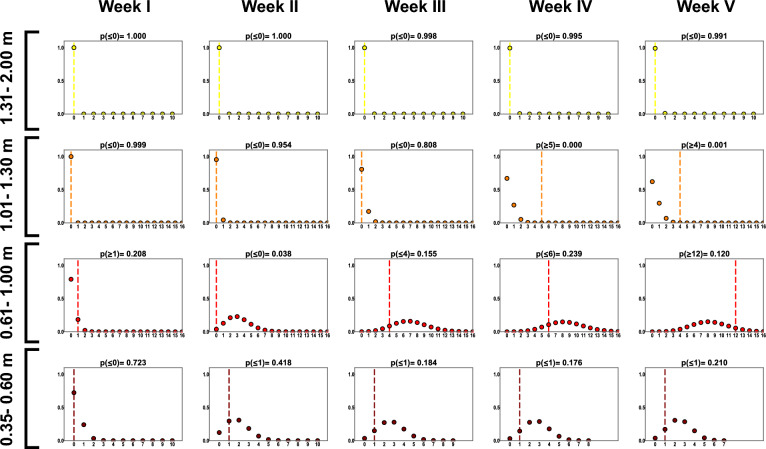
Probability mass functions generated from model predictions for type 1 experiments, representing total number of cases per 1 spatiotemporal bin of 1 week for pens grouped into four distance bins: 0.35–0.60 m, 0.61–1.00 m, 1.01–1.30 m, 1.31–2.00 m; on the x axis is the number of positive cases observed during a 1-week interval, and the y axis shows the probability. The vertical line marks the total number of cases observed for the particular bin in the experiments.

**Figure 4:**
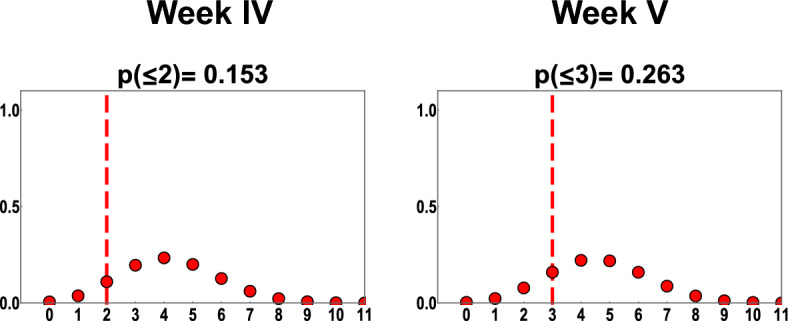
Probability mass functions generated from model predictions in type 2 experiment for group A representing total number of cases per week; on the x axis is the number of positive cases observed during a 1-week interval, and the y axis shows the probability. The vertical line marks the total number of cases observed for the particular bin in the experiments.

**Figure 5:**
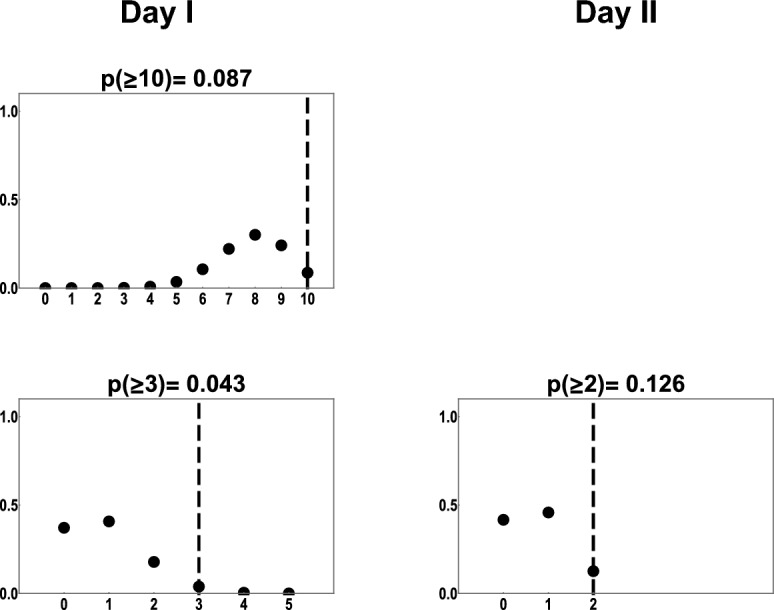
Probability mass functions generated from model predictions in type 2 experiment for group B (upper row) and C (lower row), representing total number of cases per day; on each plot the x axis is the number of positive cases observed during a 1-day interval, and the y axis shows the probability. The vertical line marks the total number of cases observed for the particular bin in the experiments.

To validate the quality of our model fit to the data we assessed the fit across both the spatial and temporal dimension by using 20 and 5 spatiotemporal bins for type 1 and type 2 experiment, respectively. For each spatiotemporal bin we calculated the total number of positive cases observed during experiments and compared these to the probability mass function for number of cases, given the number exposed, as predicted by the model. The results for the type 1 experiments are presented in Fig. 3, the results for the type 2 experiments are presented on Fig. 4 (group A) and Fig. 5 (group B and C).

We calculated a p-value for each spatiotemporal bin as the total model probability for obtaining the observed number of cases or more extreme values. For almost all spatiotemporal bins p-values are higher than 0.025. In type 1 experiments, for distance range 1.01–1.30 m, for the last two spatiotemporal bins significantly more cases were observed in the experiments than the model predicts (p < 0.025), because of that the Fisher’s combined probability test performed for all 25 spatiotemporal bins indicates significant difference between model fit and experimental data (p = 0.000665). However, once p-value for those two spatiotemporal bins are removed, the combined p-value indicates overall good fit (p = 0.166345). For a distance bin 0 m (group B and C in type 2 experiment), the fit for individual bins is consistent with experimental data (p > 0.025), but the observed outcome for all three bins is located in the right tail of distribution and the Fisher's combined probability test performed for them shows a significant difference between data and model for these 3 bins (p = 0.017891). Detailed results from Fisher’s combined probability test are provided in Supplementary Note S1.

## Discussion

Here, we presented a mechanistic model, based on the biological hypotheses of pathogen dispersal and decay, that has three parameters which are identifiable with transmission data only. Previously, a four-parameter version of the model was used (of which the decay rate was estimated independently from a survival experiment)^3^. The fourth parameter called “exposure capacity” was introduced based on the assumption that there is a limiting value of the amount of infectious material to which a host can be exposed; for modelling it means that when the exposure capacity is reached the probability of infection is constant even if accumulated amount of material increases. As there is no clear biological hypothesis underlying this assumption and we decided to start the analysis of distance dependence from the simplest mechanistic model possible, in the current work we used a model without this additional parameter. In the current model the exposure is still constrained, just as an emerging property of the balance between shedding of material on one hand and decay and diffusion on the other.

With the observations from all experiments together we were able to calibrate the model by obtaining point estimates and finite confidence intervals for all three parameters- decay rate parameter α, diffusion coefficient D and transmission rate parameter β using transmission data only. In the previous analysis^3^, the decay rate was estimated from a separate survival experiment. As we show here, identifying it together with the other two parameters from transmission data only was not possible because only one distance band was studied. This is an example of a practical unidentifiability (as opposed to structural identifiability), as it arises because the data used for parameter estimation is insufficient^37^.

As a main solution to problems with identifiability it is often recommended to take separate measurements (e.g. for exposure) and/or conduct separate small scale experiments to estimate some of the parameters independently from transmission data^38^. While sometimes it is the only possible way to obtain the estimates, it can also be problematic, especially for processes that are poorly understood.

The survival of *C. jejuni* is an example of such a poorly understood process; better understanding of the survival and transmission of *C. jejuni* in poultry was recognised as an important knowledge gap for farm level control of *C. jejuni*^39^. As many survival mechanisms could be involved, such as biofilm formation and/or viable but non-culturable states (VBNC)^40^, it is difficult to assess the limitations of designing the separate survival experiment to mimic the exact conditions of animal transmission experiments. For that reason, one of the objectives of this study was to estimate decay rate (together with remaining parameters) from transmission data only. Previously, the decay rate was estimated in the separate survival experiment to be α = 2.25 day^−1^ by fitting exponential decay curve to enumeration of *C. jejuni* in faeces^3^. Now, based on transmission data only, we find a decay rate estimate that is significantly different: α = 0.153 day^−1^ (CI 0.072–0.295). Also, our current model when fitted with fixed α = 2.25 day^−1^ is not able to reproduce the observed delay in transmission and its AIC value (503.169) is much higher than the one obtained while fitting all three parameters (490.125) (see Supplementary Note S3 for details). This difference between estimates from two different experimental setups may be explained by various hypotheses. During the separate survival experiment, the authors mimicked environmental (climate) conditions of transmission experiments and were careful to use the same type of infected animals to produce the infectious material and use the same bacterial strain; however, the estimation was based on enumerations of culturable bacteria made from samples of faeces^3^. While only culturable forms of *C. jejuni* were measured in the experiment, there is some support for the hypothesis that viable but non-culturable forms (VBNC) can also successfully colonize chickens^41–43^. Moreover, it is not well studied in what form *C. jejuni* contaminated material is dispersing spatially; a faecal sample may not be representative for the actual infectious particles that diffuse in the environment and thus were studied in transmission experiment. Most likely infectious material is dispersed in smaller particles, for example with dust or water droplets. As these small particles are likely to have different micro-conditions than big particles of faeces, the decay rate of *C. jejuni*, even in similar environmental (macro)conditions, may be different for these two situations. Additionally, when fitting an exponential decay curve to survival data, the assumption is made that each particle has the same mean probability for decay. It is possible that particles that contribute significantly to transmission between separated broilers, are able to survive in the environment much longer than the average. Further study is needed to explore which hypotheses (or combination thereof) can explain the observed difference. From a modelling perspective it could be addressed as a biphasic environmental decay^44^. Such a biphasic decay was proposed previously for *E. coli* survival modelling^45^ and it was demonstrated that including this modified survival significantly improves the model fit to the concentration data for *Cryptosporidium* and *E. coli*^44^. From an experimental perspective, methods for detection of VBNC could be included^46^ to study decay in various conditions. VBNC states occur in many bacteria; apart from campylobacter, these include *Vibrio cholera, E. coli, Salmonella enteritidis, Shigella sonnei, and Legionella pneumophila*^47^.

The spatial dispersion of infectious material produced with faeces is another poorly understood process that we aimed to understand better. Although for some diseases, with faecal-oral transmission, dispersion between separate hosts was studied experimentally^4,8^, the specific mechanisms of pathogen dispersion are not well understood. In our mechanistic model the diffusion coefficient describes how the spatial distribution of particles changes in time as the diffusion is one of the simplest way to mechanistically describe dispersal. The mechanism underlying standard diffusion is a random walk of individual particles and if many particles are involved the resulting distribution can be interpreted as a relative amount of material present at each location^23^. The estimate for the diffusion coefficient we obtained (0.013 m^2^ day^−1^; CI 0.008–0.023) as well as a delay of transmission observed in our experiments (and consistently in the fitted model) indicates that the dispersion of infectious material is relatively slow for *C. jejuni*. Most likely the spatial dissemination of material is a multistep process, influenced by factors such as caretaker movements, birds behaviour (e.g. wing flapping, water spilling), ectoparasites or ventilation. During our experiments no directional pattern was observed that could be explained by air flow (controlled in our experimental rooms), order of sampling (with inoculated broilers always being sampled last) or other caretaker actions (that were preformed following strict hygienic measures; see detailed description of experiments provided in “Methods” section). Our experimental observations altogether indicate that a direct (one-blow) air-borne transfer of *C. jejuni* carrying particles is unlikely.

Both the decay rate parameter α and the diffusion coefficient D used in the current version of the model describe well defined biological processes. The decay rate describes the process in which the infectious forms of pathogen (*C. jejuni* in our case) lose their infectivity (e.g. bacteria die) exponentially, while the diffusion coefficient describes the process of spatial dissemination of infectious material assuming the random walk as a basis of this process.

Interpretation of the transmission rate parameter β is less straightforward. It is in fact the combination of parameters describing three host dependent processes: (1) a shedding rate describing the amount of material that is produced by the infectious host; (2) an exposure rate describing how recipient hosts contact the infectious material dispersed and accumulated in the environment, and (3) a probability of becoming colonised per one unit of ingested dose describing dose response. A way to ease the interpretation of the transmission rate parameter in our model, and to aid comparison between systems, is by bringing the infectious unit on the same footing as in direct transmission models. In a direct transmission model the exposure unit is usually defined as the total exposure due to one infectious individual shedding during one timestep $\Delta t$ (usually of one day) and this exposure is taken proportional to $\frac{1\times \Delta t}{N}$ (N being the number of hosts). In models describing transmission through the environment the infectious unit can still be defined such that it corresponds to one infectious individual, according to the following recipe: starting from a well-defined state of the environment (equilibrium or clean) at the beginning of a timestep ∆t, one integrates the environmental load originating from a single infectious individual (source host) over the timestep ∆t and over the recipient exposure area. The mathematical formalism of the standardization should be developed further in future studies.

Given the fact that our model has only three estimable parameters, we believe that the model fit demonstrates a very good description of the observations. The fitted model describes satisfactorily the delay of transmission observed in the experiments, and how this delay depends on the distance between infectious (source) host and recipient hosts. When sources and recipients are housed together in an initially clean environment the delay of transmission is very small (1–2 days), and when recipient hosts are separated from the source hosts the delay of transmission is increasing with the distance of separation (from a few days of delay to no infection observed during the full observation period of 35 days). In addition, the model also describes satisfactorily that the delay was much shorter when recipient hosts distanced from the source hosts were placed in an already contaminated environment (type 2 experiments, groups A and B) than when the experiment started from a clean environment (type 1 experiments and group C of type 2 experiment). The model explains this as the result of accumulation of the infectious material in the environment to which recipient hosts are exposed, including material produced before the recipients were introduced. This all indicates that the observed delay is a result of decay and diffusion dynamics rather than a result of an overall low transmission rate (constant in time).

In the type 2 experiment we classified animals in direct contact as separated by a distance of 0 m (therefore assuming only indirect transmission) as *C. jejuni* is mainly transmitted via the faecal-oral route. Generally, for both experimental groups (A and B) the observed number of cases is slightly higher than the model predicts. This could be an indication that if recipient and source hosts are housed together additional routes of infections are present in comparison to when they are housed separately; for example sharing drinkers and feeders may contribute to transmission. As we had only a small sample size (10 broilers in group B and 5 in group C) for both pairwise groups, collection of more data could allow one to assess the effect size of potential additional sources on transmission.

Here, we used *C. jejuni* transmission in broilers as a host-parasite model system. The motivation for this was two-fold. First, *C. jejuni* is a zoonotic pathogen transmitted through the environment via the faecal-oral route that remains a major public health problem despite many control programs that have been applied^48^. To further develop intervention strategies, a better understanding of its transmission dynamics is crucial. Second, *C. jejuni* transmission in poultry is a convenient system to study small scale environmental transmission. Fast transmission dynamics (extremely short incubation period, relatively short survival in environment) reduces time of experiments, clear manifestation (high, consistent shedding) facilitates the detection and sustainability of transmission chains; while low pathogenicity in broilers is important for animal welfare^49^. As we show here, this model system is also suitable to study distance dependence of transmission: using practical between-host distances of up to 2 m enabled us to combine observations ranging from extremely fast transmission (when hosts are housed together), via slower transmission (across middle distances) to absence of transmission (across longer distances).

The experimental design presented in this paper can potentially be used to study other systems, e.g. pathogens surviving longer or shorter than *C. jejuni* or having different transmission routes. As we show here, for experiments to provide data of sufficient quality and quantity for model identifiability, the design of the experiments needs to include an informative spatiotemporal resolution. In one of the previously published experiments, we studied also the transmission of *E. coli*, a bacteria that survives in the environment much longer than *C. jejuni*^3^. Inclusion of this pathogen was not possible in subsequent experiments for practical reasons. As we have shown, the data from one of the previously published experiments alone has insufficient spatial resolution to do a similar analysis for of *E. coli* as was done here for *C. jejuni*. Further study, i.e. a full identifiability analysis, is needed to provide recommendations for the experimental design for other host–pathogen-environment systems.

By estimating all the parameters with transmission data (i.e. exposure and infection data) only, we were able to validate our mechanistic model and gain further insights into environmental transmission of *C. jejuni*. The model fit confirms that a delay (to onset) of distant transmission observed in experiments can be explained by the dynamics of decay and diffusion of infectious material. The estimated diffusion rate indicates that dispersion of infectious material for faecal-oral diseases is rather slow. Moreover, estimating the decay rate together with other parameters opens new opportunities to study the decay process of pathogens. Generally, insights obtained from transmission data for validated models can be analysed together with the data from separate pathogen survival experiments to study further mechanisms of *C. jejuni* decay in animal systems.

We formulated a methodological framework based on a parsimonious, yet mechanistic model and proved that it can be calibrated and validated with spatiotemporal transmission data. This approach does not require any prior knowledge on detailed transmission routes which are often difficult to determine. Additionally, using our methodological framework the impact of efficient intervention strategies targeted on the environmental stage of transmission, such as cleaning regimens, can be assessed quantitatively through experimental study, supported by mechanistic modelling of transmission, dispersion and decay.

## Methods

### Model

We constructed a spatiotemporal, mechanistic, yet parsimonious transmission model that takes into account the dispersion and decay of the assumed infectious material and for the probability of infection the dose response given the exposure (Eq. 2 in the “Introduction” section). Our model can be classified as individual based, where each host is described with the spatial coordinates of the area it occupies and from that, the exposure for a particular period and recipient area (occupied by recipient hosts) is calculated based on the infectious period and source area (occupied by infectious hosts). The dispersion of material is described as a diffusion process, which is based on the assumption that particles can be modelled as moving according to a random walk (each particle is making random steps through the environment, where each step has a random direction and the step length is a normally distributed random variable). The decay is described using exponential function, thus assuming that every infectious unit has the same probability of survival^50^. The instantaneous rate of infection at any location and time is given as a mathematical expression and from that infection probabilities can be calculated in any spatiotemporal resolution of choice (Eq. 2 in the “Introduction” section). This makes the model easily adaptable to transmission systems that vary in spatial organisation or temporal dynamics of exposure.

In all our experiments host position was restrained within pen boundaries, and hosts occupied an areas separated from the source by a fixed distance, so the probability of infection can be modelled based on time independent source and recipient areas. Moreover, we assumed in the model that source areas emit infectious material continuously throughout the whole shedding period.

The definition of all parameters and variables is provided in Table 2.

**Table 2:** Parameters and variables for the spatial model of environmental transmission.

Parameters		
α	[day^−1^]	Decay rate parameter
β	[day^−1^]	Transmission rate parameter
D	[m^2^ day^−1^]	Diffusion coefficient
Configuration parameters		
Q	[m^−2^ day^−1^]	Source strength
${A}_{\text{inf}}^{i}$	[m^2^]	Source area occupied by infectious host(s) *i*
${A}_{\text{exp}}^{r}$	[m^2^]	Exposure area occupied by recipient host(s) *r*
Temporal variables		
$\left({T}_{1}^{i}\text{, }{T}_{2}^{i}\right)$	[day]	Emission period of the source area *i*
$\left({t}_{1}^{r},{t}_{2}^{r}\right)$	[day]	Exposure period of the recipient area *r*

The density function of environmental load generated by a given source area ${A}_{\text{inf}}^{i}$ occupied by an infectious individual *i* is described by the following equation:

$${W}^{i}\left(\text{t, x, y, }{T}_{1}^{i}\text{, }{T}_{2}^{i}\text{, }{A}_{inf}^{i}\right)=\left\{\begin{array}{ll}0& t<{T}_{1}^{i}\\ Q{\int }_{{T}_{1}^{i}}^{t}{\iint }_{{A}_{\text{inf}}^{i}}\frac{1}{4\pi D\left(t-\tau \right)}{\text{exp}}\left[-\alpha \left(t-\tau \right)-\frac{{\left(x-{x}_{i}\right)}^{2}+{\left(y-{y}_{i}\right)}^{2}}{4D\left(t-\tau \right)}\right]d{x}_{i} \, d{y}_{i} \, d\tau & {T}_{1}^{i}\le t\le {T}_{2}^{i}\\ Q{\int }_{{T}_{1}^{i}}^{{T}_{2}^{i}}{\iint }_{{A}_{\text{inf}}^{i}}\frac{1}{4\pi D\left(t-\tau \right)}{\text{exp}}\left[-\alpha \left(t-\tau \right)-\frac{{\left(x-{x}_{i}\right)}^{2}+{\left(y-{y}_{i}\right)}^{2}}{4D\left(t-\tau \right)}\right]d{x}_{i} \, d{y}_{i} \, d\tau & {T}_{2}^{i}<t\end{array}\right.$$

where the ${W}^{i}$ definition distinguishes three cases: (1) if the source is not yet emitting (time of observation is earlier than start of emission) ${W}^{i}$ is equal to 0; (2) if the source has started continuous emission (time of observation is somewhere during the emission period) the environmental load is represented as diffusion with continuous source and decay and accounts for all the material that was released from the start of emission to the time of observation; as discussed in more detail in van Bunnik et al.^3^ the part ${\text{exp}}\left[-\alpha \left(t-\tau \right)\right]$ in the integrand takes into account the decay, the part $\frac{1}{4\pi D\left(t-\tau \right)}{\text{exp}}\left[-\frac{{\left(x-{x}_{i}\right)}^{2}+{\left(y-{y}_{i}\right)}^{2}}{4D\left(t-\tau \right)}\right]$ the 2 dimensional diffusion (see van Bunnik et al.^3^ for derivation), the integrals over ${x}_{i}$ and ${y}_{i}$ take into account the emission from the source area ${A}_{\text{inf}}^{i}$, and the integral over $\tau$ takes into account all material release times until the time of observation; (3) if the source has already stopped emitting (time of observation is after emission period) ${W}^{i}$ accounts for all the material that was released in the past during the emission period and its decay and further diffusion up to the time of observation. We assume that the source area is emitting infectious material as soon as at least one host housed inside the area starts shedding. Q is a factor that scales the source strength level to account for differences in density of infectious host per square meter of area, for example in situations when infectious host are occupying areas of various sizes.

For a given recipient area ${A}_{\text{exp}}^{r}$ that is exposed to multiple sources (more than one source area present), the source term is implemented as a set ${\left\{{T}_{1}^{i}, {T}_{2}^{i},{A}_{\text{inf}}^{i} \right\}}_{I}$, and Eq. 2 (in the “Introduction” section) that describes the probability of infection for particular recipient host *r* turns into:

$${\mathrm{P}}_{\text{inf}}\left({t}_{1}^{r},{t}_{2}^{r}\text{, }{A}_{\text{exp}}^{r}\text{,}{ \left\{{T}_{1}^{i}, {T}_{2}^{i},{A}_{\text{inf}}^{i} \right\}}_{I}\right)=1-\mathrm{Exp}\left[-\upbeta {\sum }_{I }{\int }_{{t}_{1}^{r}}^{{t}_{2}^{r}}{\iint }_{{A}_{\text{exp}}^{r}}{W}^{i}\left(\text{t, x, y, }{T}_{1}^{i}\text{, }{T}_{2}^{i}\text{, }{A}_{\text{inf}}^{i}\right)d{A}_{\text{exp}}^{r} dt\right],$$

where ${\sum }_{I }$ is the sum over the set of source areas.

### Experimental data

**Figure 6:**
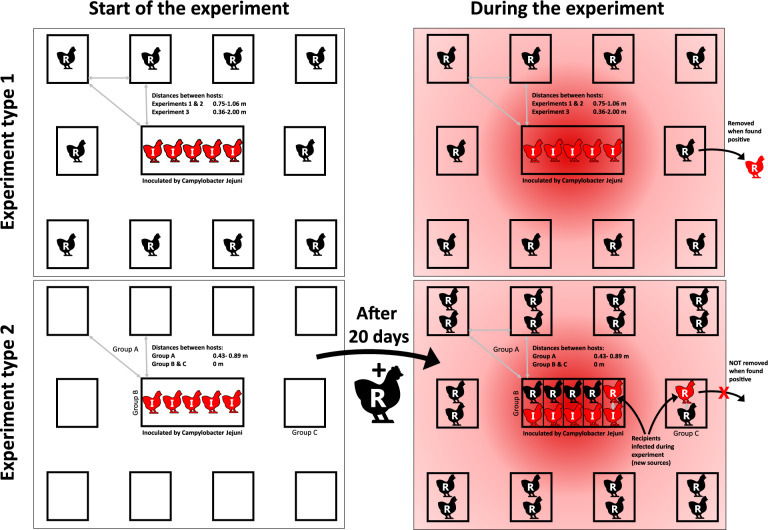
Schematic representation of experimental design for 5 experiments studying transmission of *C. jejuni* between broilers spatially separated by various distances (0–2 m): three type 1 experiments where exposure started in a clean environment and where the newly infected recipients broilers were immediately removed, and one type 2 experiment, where exposure started in a contaminated environment and where the newly infected recipients broilers were not removed and considered new sources of infection. The detailed description of the experiments is provided in the manuscript.

Spatiotemporal data used to fit the model were obtained in series of experiments on indirect transmission of *C. jejuni* in broilers. In total, data from five experiments, each with multiple groups, were included. A schematic representation of the experimental design is provided on Fig. 6

The animal experiments and associated procedures were in accordance with the national regulations on animal experimentation and the project licenses were approved by the Dutch Central Authority for Scientific Procedures on Animals (CCD) (permit numbers for previously unpublished experiments: AVD4010020172784 for experiment 4; AVD4010020198586 for experiment 5). Here we follow the ARRIVE guidelines; the detailed description of previously unpublished experiments is provided below (in the “Detailed description of transmission experiments” section).

All experiments lasted 35 days and the status of broilers was recorded daily by testing cloacal swabs for the presence of *C. jejuni*. We used data from four type 1 experiments, where exposure of the recipient from the source hosts started in a clean (i.e. *C. jejuni* free) environment, and one type 2 experiment where recipient hosts were placed in experimental room 20 days after the source animals were inoculated with *C. jejuni*. The schematic spatial organisation of all experimental rooms is provided as supplementary figures (Figs. S7–S15).

All type 1 experiments had a similar design. In this analysis we included rooms were five source broilers were orally inoculated with *C. jejuni* on day 0 and placed in one pen situated in the centre of the experimental room. On the same day, *C. jejuni* negative recipient broilers were individually placed in smaller recipient pens spatially separated from the source pen. For experiments 1–3 the spatial setup was similar, the source and recipient pens were approx. 0.75–1.06 m apart in each experimental room; as detailed between-pen distance measurements were not collected during those experiments, coordinates used in the model likelihood calculation were determined based on assumption that all rooms had identical, symmetrical design. For experiment 4 spatial setup was different, border to border distance between pens ranged from 0.35 to 2.00 m and the pens were divided over 6 experimental rooms; for this experiment between-pen distances in all rooms were recorded such that pen coordinates could be calculated for each pen separately for use in the model likelihood calculation.

All rooms in the experiments 1–3 had 10 recipient pens. Experiments 1 and 2 were identical in design and each had animals housed in two separate experimental rooms. In experiment 3 animals were housed in eight experimental rooms; in four rooms 5 inoculated broilers were used; in the remaining four rooms 20 inoculated broilers were used and these were excluded from analysis presented here. In two out of four of the included rooms, the broilers in the central pen were also inoculated with *E.coli*. It has been shown in van Bunnik et al.^3^ that *E.coli* infection is not likely to influence *C. jejuni* results. In the current analysis, we therefore included the *C. jejuni* data of these rooms. In experiment 4 there were 6 experimental rooms, four with 10 recipient pens and two with 4 recipient pens (separated from the source by larger distances). In all type 1 experiments recipient broilers that were detected positive for *C. jejuni* during the experiment were immediately removed from the experimental room to make sure their contribution to the probability of infection was minimal.

The last experiment (experiment 5) was a type 2 experiment and had a different design than the previous ones. The initial conditions were similar to previous experiments: five broilers per experimental room were inoculated with *C. jejuni* and placed together in a central pen at day 0. On day 20, the central pen was divided into 5 adjacent sub-pens similar in size, each housing one of the inoculated broilers. On the same day, recipient broilers were placed in the experimental room. Three groups of recipients were distinguished: group A consisted of ‘distant’ recipients placed in ten pens separated from the central pen by 0.43–0.89 m; each of these pens housed a pair of recipient broilers; Group B was a pairwise type of recipients for which *C. jejuni* free broilers were added to the five central sub-pens, so each sub-pen was housing one inoculated and one recipient broiler; this group was included to study the transmission when the distance between source and recipient host was assumed to be 0 m. Group C, classified after the experiment, was also a pairwise type of recipients, but housed in distanced pens; the recipient was classified to group C after their pen mate started shedding *C. jejuni* during the experiment (becoming the source of the infectious material).

Both experimental rooms in experiment 5 had a similar spatial setup and the size of the central pen was bigger than the size used in type 1 experiments; again all between-pen distances were measured. In each room 5 central sub-pens and 10 recipient pens were present. The source broilers in this experiment were also inoculated with *Salmonella enterica* serovar Enteritidis. Previous transmission experiments, where broilers were inoculated with the same bacterial strains, showed that it is unlikely that inoculation with *Salmonella* had an influence on *C. jejuni* transmission^51,52^. In the current analysis, we used the *C. jejuni* data only, as we estimated only parameters for *C. jejuni* transmission. In contrast to previous experiments, recipient broilers that were detected positive for *C. jejuni* were not removed during the experiment.

### Likelihood formulation and parameter estimation

To estimate parameters for the spatiotemporal model with data from transmission experiments several assumptions were made. We assumed that once any broiler is detected positive for *C. jejuni* it starts to shed pathogen continuously until the end of the study period. This was based on the fact that in our experiments all broilers, detected to be positive for *C. jejuni* once and not removed from experiment, remained positive for the rest of the experimental period. This assumption applies for all inoculated broilers in all experiments and all recipient broilers in experiment 5. For experiments 1–4 recipient broilers who became infected were removed immediately when detected positive; considering the fact that the shedding periods of those individuals were short and diffusion of *C. jejuni* is relatively slow, their contribution to the probability of infection up until the end of the study period was considered to be negligible and not included in likelihood function (see Supplementary Note S4 for details). These approximations allowed us to proceed with a much simpler likelihood function.

In the model, sources of infectious material are described in terms of areas that *C. jejuni* shedding hosts occupy. For all experiments the central pen housing inoculated broilers was considered source area since the day of inoculation, considering the short incubation time of *C. jejuni*; the source strength for all central pen areas (occupied by multiple source broilers) was modelled as constant in time. For the type 2 experiments, where positive recipient hosts were not removed, newly positive broilers were included into the model as new source areas from the day of detection onwards. As the source areas had a different size in type 1 as compared to type 2 experiments and additional source areas were present in the latter, we standardized the source for all experimental rooms by using the host density (number of broilers per square meter) as a source scaling factor for each particular source area.

In contrast to type 1 experiments, where recipient broilers were housed individually, in type 2 experiments each pen housed a pair of broilers. For model fitting we assumed that these paired broilers do not compete for infectious material and treat them as independent recipients.

To estimate the model parameters, we maximized the likelihood function given by:

$${\prod }_{{\left\{ {t}_{1}^{r},{t}_{2}^{r}\text{, }{A}_{\text{exp}}^{r} \right\}}_{R{\text{esc}}}}\left[{1-\mathrm{P}}_{\text{inf}}\left({t}_{1}^{r},{t}_{2}^{r}\text{, }{A}_{\text{exp}}^{r}\text{,}{ \left\{ {T}_{1}^{i}, {T}_{2}^{i},{A}_{\text{inf}}^{i} \right\}}_{I}\right)\right]\cdot {\prod }_{{ \left\{ {t}_{1}^{r},{t}_{2}^{r}\text{, }{A}_{\text{exp}}^{r} \right\}}_{R{\text{inf}}}}\left[\left({1-\mathrm{P}}_{\text{inf}}\left({t}_{1}^{r},{t}_{2}^{r}-1\text{, }{A}_{\text{exp}}^{r}\text{,}{ \left\{ {T}_{1}^{i}, {T}_{2}^{i},{A}_{\text{inf}}^{i}\right\}}_{I}\right)\right)\cdot {\mathrm{P}}_{\text{inf}}\left({t}_{2}^{r}-1,{t}_{2}^{r}\text{, }{A}_{\text{exp}}^{r}\text{,}{ \left\{ {T}_{1}^{i}, {T}_{2}^{i},{A}_{\text{inf}}^{i} \right\}}_{I}\right)\right].$$

where ${\left\{ {t}_{1}^{r},{t}_{2}^{r}\text{, }{A}_{\text{exp}}^{r} \right\}}_{R{\text{esc}}}$ is a set of data of all recipients that escaped from infection during time of exposure from ${t}_{1}^{r}$ to ${t}_{2}^{r}$, while ${\left\{ {t}_{1}^{r},{t}_{2}^{r}\text{, }{A}_{\text{exp}}^{r} \right\}}_{R{\text{inf}}}$ is a set of data of all recipients that were found positive during time of exposure from ${t}_{1}^{r}$ to ${t}_{2}^{r}$.

The data input system, automatic likelihood formulation, three-step maximization procedure, statistics performed for model validation and all addition calculations were programmed in Mathematica 12.0^53^; the code is available in Zenodo repository^54^. In detail, the log-likelihood function was formulated jointly for the spatiotemporal *C. jejuni* transmission data from all experimental rooms from all experiments. In the maximization procedure, all spatial integrals (integrals over source areas as well as recipient area) in the expression for the probability of infection were analytically solved using the Integrate function, which significantly reduced computation time. Temporal integrals were solved numerically using the NIntegrate function. The log-likelihood function was maximized for all 3 parameters using a three-step procedure. First, a univariate profile likelihood was calculated for the diffusion coefficient D using NMaximize function. We used the D profile likelihood as a first step in our optimization, because the maximization with fixed D was the fastest and most prone to find only global maxima. Second, based on the generated D profile likelihood, we manually set suitable constraints for all three parameters within which the NMaximization function maximized their values. These constraints were applied to speed up the calculations and prevent the NMaximization function from returning only local maximum. Lastly, the univariate profile likelihoods were obtained for remaining two parameters (α and β), using the same procedure as for the parameter D, including the application of constraints when needed to prevent the NMaximization function from returning local maxima. Confidence bounds for parameters were obtained from the corresponding profile likelihoods using the likelihood ratio test; if needed additional points were calculated in regions near the bounds to achieve the desired accuracy of 0.001.

### Statistical analysis of model fit

To validate the model, we aggregated model fits and experimental data into spatiotemporal bins. For type 1 experiments, we defined 4 distance bins (0.35–0.60 m, 0.61–1.00 m, 1.01–1.30 m, 1.31–2.00 m) and these were divided further into 5 spatiotemporal bins of 1 week each, which gave us total of 20 spatiotemporal bins. For type 2 experiment, in group A (distant recipients) there was only one distance bin because all but one of the pens were separated by 0.61–1.00 m; one pen was separated by 0.43 m but was also included in the same bin for statistical analysis. The distance bin was divided into two spatiotemporal bins of 1 week. For group B and group C (pairwise groups) there was one distance bin of 0 m divided into 1 or 2 spatiotemporal bins of 1 day, as for these groups the dynamics of transmission was much faster.

For each spatiotemporal bin we compared the sum of positive cases observed in experiments to the probability of infection calculated from model fit obtained for parameter values that globally maximized the likelihood (point estimates). The probability of infection for each bin was calculated as probability of being infected anytime during this particular bin. In group A (distant recipients) in type 2 experiment, a pair of broilers occupied each pen and the probability of infection was defined as probability of observing at least one of the two broilers becoming colonized. We note that the probabilities of being colonised in general differed between the individual pens belonging to the spatiotemporal bin, as the probability varied depending on distance and position to the source and it was calculated using recipient (pen) areas coordinates. Therefore, the probability mass function (PMF) for number of colonised pens in each spatiotemporal bin was calculated using the Poisson Binomial distribution, being the distribution for the sum of independent Bernoulli distributed variables with varying p. In the calculations we used the discrete Fourier transform formula to calculate PMF^55^, as in some of the bins the number of cases was (much) bigger than 10 and those were therefore non-computable from exact PMF formula.

Next, from the PMFs we calculated the p-value as the probability of observing the particular experimental outcome or more extreme values; for outcomes occurring in the first half of the distribution this meant integrating over the left tail of the distribution and for outcomes in the second half it meant integrating over the right tail. P-values lower than 0.025 were considered as significant.

To further diagnose the model fit we used Fisher's combined probability test to combine p-values generated for spatiotemporal bins.

### Detailed description of transmission experiments

Results from experiments 1–3 were previously published, thus their description can be found in van Bunnik et al.^8^ (experiments 1 and 2) and van Bunnik et al.^3^ (experiment 3).

#### Description of experiment 4

##### Experimental design

The experiment was carried in six experimental rooms. Five broilers inoculated with *C. jejuni* were housed together in one pen in the centre of every experimental room (a separate climate-controlled room in an experimental facility of the Wageningen Bioveterinary Research). In four rooms 10 recipient animals and in two rooms 4 recipient animals (all *C. jejuni* negative at the beginning of experiment) were housed individually in pens placed at various distances (see Figs S8–S13 for spatial design and pen coordinates) from the central pen.

During the experiment, all source and recipient animals were sampled by means of a cloacae swab. To confirm their Campylobacter status, the swabs were tested for the presence of *C. jejuni*. If a tested recipient animal was found *C. jejuni* positive it was immediately removed from the experiment, euthanised and the cecum was removed and tested for *C. jejuni*. A broiler was considered infected when both swab and caecal sample were found positive for *C. jejuni*; this was the case for all positive recipients. The experiment ended 35 d post inoculation. All remaining source and recipient animals (that had not been found *C. jejuni* positive until that moment) were euthanized after which the cecum was removed and a caecal sample was tested for *C. jejuni*.

##### Animals and housing

One-day-old broilers (type Ross 308, females) were obtained from a commercial hatchery. Only female broilers were used to prevent overgrowing of broilers and health problems attributed to that.

88 chicks were housed together in auxiliary room from day − 14 to day − 2. At day − 6 and − 2, cloacal swabs taken from each chick were tested to confirm the absence of *C. jejuni*.

On day − 2, 78 chicks were randomly distributed to six experimental rooms for the transmission experiment. Remaining reserve broilers were euthanised. Four rooms contained 5 source animals housed together in one central pen and 10 recipient animals individually housed in 10 pens surrounding the central pen as shown in Figs. S8–S11. The other two rooms contained 5 source animals housed together in one central pen and 4 recipient animals individually housed in 4 pens surrounding the central pen as shown in Figs. S12 and S13. All animals were housed on wood shavings and the drinking water was supplied through a nipple drinking system. In each setup, the drinking nipples in the pens on the long sides of the area were supplied from one common water container, and the central pen had a separate drinking water supply. This precluded transmission via a shared drinking water system. Before the start of the experiment, all experimental rooms were cleaned and disinfected with formaldehyde. Subsequently, samples were taken from inside the room to check for the absence of *C. jejuni*.

##### Inoculation

At day 0 source broilers were inoculated with 1 ml of the inoculum containing *Campylobacter jejuni*, dose 5.4 × 10^6^ CFU/ml applied to the crop. For inoculation with *C. jejuni*, the *C. jejuni* strain 356 was used. The strain was cultured in hearth infusion broth (WBVR BM332) microaerobically at 37 °C, overnight, and diluted in buffered peptone water to obtain the intended inoculation dose [∼1 × 10^6^ colony forming units (CFU)/mL]. The precise concentration (CFU per milliliters) of *C. jejuni* in the administered inoculum was determined by plating serial tenfold dilutions on modified cefoperazone charcoal deoxycholate agar (mCCDA; WBVR BM322).

##### Sampling and detection

To check their status all broilers were tested by taking cloacae swabs. The sampling scheme is provided in Table 3.

**Table 3:** Sampling scheme for experiment 4.

Day post inoculation	
− 6	Cloacal swabs of all broilers
− 2	Cloacal swabs of all broilers
0	Cloacal swabs of all broilers (before inoculation) Inoculation
0–6	Cloacal swabs of all broilers
7–11	Cloacal swabs of all recipients
12	Cloacal swabs of all broilers
13–18	Cloacal swabs of all recipients
19	Cloacal swabs of all broilers
20–25	Cloacal swabs of all recipients
26	Cloacal swabs of all broilers
27–32	Cloacal swabs of all recipients
33	Cloacal swabs of all broilers
35	Cloacal swabs of all recipients; caecal samples from all remaining broilers

Swab samples were collected using sterile swabs (sterile plain dry swabs; Copan Diagnostics, Inc.). For *C. jejuni*, swabs were directly plated on mCCDA (WBVR BM332), incubated microaerobically at 41.5 °C and examined for the presence of *C. jejuni* after 24 and 48 h. After streaking on mCCDA, the swab was placed in Preston enrichment medium [nutrient broth no. 2, Oxoid CM0067 with Campylobacter selective supplement (Oxoid SR0204E) and Campylobacter growth supplement (Oxoid SR0232E)], and incubated microaerobically at 41.5 °C for 24 h. After incubation, it was plated on mCCDA and incubated microaerobically at 41.5 °C, and examined for the presence of *C. jejuni* after 24 and 48 h. Confirmation of suspect colonies was done by MALDI (Biotyper®, Bruker).

At the end of experiment caecal samples were collected from all broilers and tested for *C. jejuni* using the same culturing methods.

##### Humane endpoints and euthanasia

Disease was not expected to arise as a consequence of infection, because the bacteria which were used, normally do not lead to clinical signs nor to mortality in broilers.

The humane endpoints were defined as follows: 1) not being able to stand up; 2) not being able to eat or drink; 3) severe depression (hardly any response on stimuli); 4) other severe discomfort.

The broilers were observed twice a day. During the experiment no animal reached any of the humane endpoints.

Euthanasia methods are listed in Table 4.

**Table 4:** Euthanasia methods for experiment 4.

Animal	Method
Broilers < 250 g	Cervical dislocation
Broilers 250–1000 g	Sedation (Xylazine and Ketamine) followed by cervical dislocation
Broilers > 1000 g	Sedation (Xylazine and Ketamine) followed by T61 admission

For a sedation 10 mg/kg Xylazine and 30 mg/kg Ketamine was applied IM.

##### Hygienic measures

To prevent animal caretakers from acting as a vector of transmission between experimental rooms, strict hygienic measures were used during the entire experiment. Clean coveralls were used at every entry into the experimental rooms. A pair of boots was dedicated to each room, cleaned on entering and exiting it by means of wading through a chlorinated bath. To prevent direct transport from one bird to the next bird, sterile gloves were changed between handling individual animals. The same order of sampling and movement direction within a room during sampling and welfare checks was followed during the full experimental timespan. Inoculated animals were always sampled last.

#### Description of experiment 5

##### Experimental design

Five groups of broilers were used in this experiment; half of each group was housed in experimental room 1 and half in room 2. Ten broilers from source broilers group were inoculated with *C. jejuni* and *Salmonella* Enteritidis. Five of inoculated animals were housed together in one pen in the centre of each experimental room from day 0 to day 20 (a separate climate-controlled room in an experimental facility of the Wageningen Bioveterinary Research). From day 20 onwards, central pen was divide into 5 sub-pens each was housing one broiler from source group and one from non-inoculated (*C. jejuni* negative) direct recipient group (10 in total); in both rooms 20 non-inoculated (*C. jejuni* negative) animals from indirect recipient groups were housed in pairs in 10 pens surrounding this central pen placed at various distances (see Figs. S14 and S15 for spatial design and pen coordinates).

During the experiment, all source and recipient animals were sampled by means of a cloacae swab. To confirm their *C. jejuni* and *Salmonella* Enteritidis status, the swabs were tested for the presence of both bacteria. Recipients positive for *C. jejuni* were not removed from experimental room as also Salmonella transmission was studied during the experiment. Broiler was considered infected by *C. jejuni* when swab samples were found positive for three consecutive days, and assumed to be infected from the day when first positive sample was recorded. The experiment ended 35 d post inoculation. All source and recipient animals were euthanized and the cecum was removed and caecal sample was tested for *C. jejuni*.

##### Animals and housing

One-day-old broilers (type Ross 308, females) were obtained from a commercial hatchery. Only female broilers were used to prevent overgrowing of broilers and health problems attributed to that.

After arrival at day − 14 all 66 chicks were housed together in auxiliary room in the communal pen. At day − 6 they were randomly assigned to experimental group: 10 to source group, 10 to direct recipient groups (I or II), 40 to indirect recipient groups (I or II) and 6 to replacement broiler groups (I or II).

Three rooms were used during experiment: 1 auxiliary room and 2 experimental rooms. The auxiliary room had 3 pens: one communal pen and two marked pens: I and II. The housing scheme for each group is described in Table 5.

**Table 5:** Housing scheme for experiment 5.

Day	
− 14	All chicks placed together in auxiliary room in the communal pen
− 6	Broilers randomly assigned to experimental group (source broiler, direct recipient I or II, indirect recipient I or II and replacement broiler I or II)
− 2	Source broilers moved to 2 experimental rooms (5 broilers per room): housed together in one pen in the centre of the room Remaining broilers moved to one of two marked pens in auxiliary room: groups with number I to pen I and groups with number II to pen II
20	The central pen divided into 5 sub-pens through placement of four meshes. The source broilers, housed before in the central pen, are divided into the 5 sub-pens. In each sub-pen a source broiler is housed together with one direct recipient taken from marked pen I or II Indirect recipients placed in distanced pens in pairs: one indirect recipient from pen I and one from pen II

All animals were housed on wood shavings and the drinking water was supplied through a nipple drinking system. In each setup, the drinking nipples in the pens on the long sides of the area were supplied from one common water container, and the central pen had a separate drinking water supply, after day 20 each central sub-pen had separate water supply. This precluded transmission via a shared drinking water system. Before the start of the experiment, all experimental rooms were cleaned and disinfected with formaldehyde. Subsequently, samples were taken from areas inside the room to check for the absence of *C. jejuni* and *Salmonella* Enteritidis.

Additionally, from day 0 to day 20 small pilot experiment was conducted to study the detection of *C. jejuni* from environmental samples. In both rooms the sampling board covered with wooden shavings was placed in pseudo-pen between pens 2–3. The board was removed before any recipients were placed in experimental room.

##### Inoculation

At day 0 source broilers were inoculated with 1 ml of the inoculum which was a mixture (1:1) of *Campylobacter jejuni*, dose 6.0×10^5^ CFU/ml and *Salmonella* Enteritidis, dose 1.2×10^5^ CFU/ml applied to the crop.

For inoculation with *C. jejuni*, *the C. jejuni* strain 356 was used. The strain was cultured in hearth infusion broth (WBVR BM332) (microaerobically, 37 °C, overnight) and diluted in buffered peptone water to obtain the intended inoculation dose [∼1 × 10^6^ colony forming units (CFU)/mL]. The precise concentration (CFU per milliliters) of *C. jejuni* in the administered inoculum was determined by plating on modified cefoperazone charcoal deoxycholate agar (mCCDA; Oxoid CM 793) with selective supplement (Oxoid CM 155) before and after the inoculation of the animals.

For inoculation with *Salmonella* Enteritidis, a nalidixic resistant (MIC > 128 mg/l) *Salmonella enterica* serovar Enteritidis phage type 4 was used. The strain was cultured on Heart Infusion Agar with 5% Sheep Blood (HIS, WBVR BM20) (37 °C, overnight) and next diluted in buffered peptone water to obtain the intended inoculation dose [∼1 × 10^5^ colony forming units (CFU)/mL]. The precise concentration (CFU per milliliters) of *Salmonella* Enteritidis in the administered inoculum was determined by plating serial dilutions on HIS (WBVR BM20) before and after the inoculation of the animals.

##### Sampling and detection

To check their status all broilers were tested by taking cloacae swab. The sampling scheme is provided in Table 6.

**Table 6:** Sampling scheme for experiment 5.

Day post inoculation	
− 6	Cloacal swabs of all broilers
− 2	Cloacal swabs of all broilers
0	Inoculation
1–6	Cloacal swabs of all source broilers
14	Cloacal swabs of all source broilers
20	Cloacal swabs of all broilers (before relocation)
21–24	Cloacal swabs of all broilers
25–27	Cloacal swabs of all recipients
28	Cloacal swabs of all broilers
34	Cloacal swabs of all recipients
35	Cloacal swabs of all broilers; caecal samples from all broilers

Swab samples were collected using sterile swabs one swab for each bacteria (sterile plain dry swabs; Copan F155CA). For *C. jejuni*, swabs were directly plated on mCCDA (WBVR BM332), incubated microaerobically at 41.5 °C for 48 h and examined for the presence of *C. jejuni* after 24 and 48 h. After streaking on mCCDA, the swab was placed in Preston enrichment medium [nutrient broth no. 2, Oxoid CM0067 with Campylobacter selective supplement (Oxoid SR0204E) and Campylobacter growth supplement (Oxoid SR0232E)], and incubated microaerobically at 41.5 °C for 24 h. After incubation, it was plated on mCCDA and incubated microaerobically at 41.5 °C, and examined for the presence of *C. jejuni* after 24 and 48 h. Confirmation of suspect colonies was done by MALDI (Biotyper®, Bruker).

For *Salmonella* enteritidis, swabs were incubated in Buffered Pepton Water for 18 h, from which 0,1 ml was plated on MRSV (WBVR 334), incubated at 37 °C for 24 h and plated out on XLD and BGA, both added with 100 ppm naladixic acid (in-house prepared). After 24 h of incubation, plates were examined for the presence of *Salmonella* Enteritidis. Suspected cultures were confirmed by MALDI typing (Biotyper®, Bruker).

At the end of experiment caecal samples were collected from all the broilers and tested for *C. jejuni* using the same culturing methods.

##### Humane endpoints and euthanasia

Disease was not expected to arise as a consequence of infection, because the bacteria which were used, normally do not lead to severe clinical signs nor to mortality in broilers. The humane endpoints were defined as follows: (1) not being able to stand up; (2) not being able to eat or drink; (3) severe depression (hardly any response on stimuli).

The broilers were observed twice a day. During the experiment no animal reached any of the humane endpoints.

Euthanasia methods are listed in Table 7.

**Table 7:** Euthanasia methods for experiment 5.

Animal	Methods
Broilers < 250 g	Cervical dislocation
Broilers 250–1000 g	Sedation (Xylazine and Ketamine) followed by cervical dislocation
Broilers > 1000 g	Sedation (Xylazine and Ketamine) followed by T61 admission

For a sedation 10 mg/kg Xylazine and 30 mg/kg Ketamine was applied IM.

##### Hygienic measures

To prevent animal caretakers from acting as a vector of transmission between experimental rooms, strict hygienic measures were used during the entire experiment. Clean coveralls were used at every entry into the experimental rooms. A pair of boots was dedicated to each room, cleaned on entering and exiting it by means of wading through a chlorinated bath. To prevent direct transport from one bird to the next bird, sterile gloves were changed between handling individual animals. The same order of sampling and movement direction within a room during sampling and welfare checks was followed during the full experimental timespan. Inoculated animals were always sampled last.

### Supplementary Information

Supplementary Information 1.

Supplementary Information 2.

## Data Availability

The authors declare that the input experimental data supporting the findings of this study are available within the paper and its supplementary information files, and in Zenodo via the following link: 10.5281/zenodo.5565053^54^.
