# Supplementary material for: Combining a parsimonious mathematical model with infection data from tailor-made experiments to understand environmental transmission
Source: Sci Rep. 2023 Aug 10;13:12986. doi: 10.1038/s41598-023-38817-z (PMC10415373; doi:10.1038/s41598-023-38817-z)
Supplement: Supplementary file 2 — Supplementary Information 2. [file 41598_2023_38817_MOESM2_ESM.docx]

**Supplementary Information**

Combining a parsimonious mathematical model with infection data from tailor-made experiments to understand environmental transmission

Anna M. Gamża^1,2^, Thomas J. Hagenaars^2^, Miriam G.J. Koene^2^, Mart C.M. de Jong^1^

1. Quantitative Veterinary Epidemiology, Wageningen University & Research, 6708 PB Wageningen, The Netherlands
2. Wageningen Bioveterinary Research, Wageningen University & Research, 8221 RA Lelystad, the Netherlands

Anna M. Gamża; e-mail: anna.gamza@ed.ac.uk

Thomas J. Hagenaars; e-mail: thomas.hagenaars@wur.nl

Mart C.M. de Jong; e-mail: mart.dejong@wur.nl

Table of Contents

[Supplementary Note 1: Fisher's combined probability test for spatiotemporal bins 2](#_Toc134394606)

[Supplementary Note 2: Model (un)identifiability from transmission data on a single distance band only 3](#_Toc134394607)

[Supplementary Note 3: Model fit and validation for fixed decay rate α=2.25 day^-1^ 5](#_Toc134394608)

[Supplementary Note 4: Additional analysis 10](#_Toc134394609)

[Supplementary figures S7 to S15: Spatial organization of experimental rooms 14](#_Toc134394610)

[Supplementary References 19](#_Toc134394611)

**Other supplementary materials for this manuscript include the following:** Supplementary dataset 1

Supplementary Note 1: Fisher's combined probability test for spatiotemporal bins

The combined p-value for Fisher's combined probability test for p-values of all the spatiotemporal bins from all experiments was: p=0.000637.

The combined p-value for Fisher's combined probability test for p-values of all the spatiotemporal bins from all experiments, except for p-values smaller than 0.0125 that were removed from the analysis (distance bin 3, week 4 & week 5) was: p=0.166430.

P-values for all spatiotemporal bins and combinations of thereof are presented in Table S1 and S2.

Table S1. P-values for all spatiotemporal bins for type 1 experiments and p-values for Fisher's combined probability test (FCP) calculated for combinations of the spatiotemporal bins of 1 week for pens grouped into four distance bins: bin 1: 0.35-0.60 m, bin 2: 0.61-1.00 m, bin 3: 1.01-1.30 m, bin 4: 1.31-2.00 m.

|  | Week 1 | Week 2 | Week 3 | Week 4 | Week 5 | FCP | FCP with removal^1^ |
| --- | --- | --- | --- | --- | --- | --- | --- |
| Bin 4 | 1.000000 | 0.999861 | 0.998330 | 0.994730 | 0.990828 | **1.000000** | **1.000000** |
| Bin 3 | 0.999352 | 0.954489 | 0.808321 | 0.000046 | 0.001221 | **0.000192** | **0.997586** |
| Bin 2 | 0.207664 | 0.038413 | 0.154957 | 0.238544 | 0.119903 | **0.024861** | **0.024861** |
| Bin 1 | 0.723084 | 0.418276 | 0.184279 | 0.175687 | 0.209769 | **0.260686** | **0.260686** |
| FCP | **0.875264** | **0.399545** | **0.479563** | **0.000927** | **0.007696** | **0.004953** |  |
| FCP with removal^1^ | **0.875264** | **0.399545** | **0.479563** | **0.384611** | **0.286783** |  | **0.591502** |

1. p values smaller than 0.025 removed from analysis (bin 3, week 4 & week 5)

Table S2. P-values for all spatiotemporal bins for type 2 experiment with p-values for Fisher's combined probability test (FCP) calculated for combinations of the spatiotemporal bins of 1 week for group A and of 1 day for groups B & C .

|  | Week 4 | Week 5 | FCP |
| --- | --- | --- | --- |
| Group A | 0.152642 | 0.263318 | **0.169377** |
|  | **Day 1** | **Day 2** | **FCP** |
| Group B | 0.086540 |  | **0.086540** |
| Group C | 0.043229 | 0.125820 | **0.033799** |
| FCP | **0.024648** | **0.125820** | **0.017891** |

Supplementary Note 2: Model (un)identifiability from transmission data on a single distance band only

In van Bunnik et al.^1^data from *C. jejuni* and *E.coli* transmission experiments between broilers separated by either 0.75 or 1.06 m were used to estimate three out of four parameters (for a model version with one additional parameter: exposure capacity). The remaining parameter, the decay rate parameter α was estimated from data obtained in a separate survival experiment in which the concentration of culturable forms of *C. jejuni* and *E.coli* was measured in faeces collected daily during a time period after removal of the broilers that produced the faeces ^1^. This survival experiment, was conducted in an experimental room identical to the rooms used in the transmission experiments with the same environmental conditions (controlled temperature and air flow)^1^. The diffusion coefficient was assumed to be the same for both bacteria ^1^.

To investigate if the three-parameter version of the model, i.e. the model that we present in the main manuscript, is identifiable with data of only a single distance band (separated by either 0.75 or 1.06 m), we fitted our model to the previously published data of *C. jejuni*. We estimated all three parameters simultaneously from the data published in van Bunnik et al. ^1^ (including only data on *C. jejuni* transmission for experiments that started with 5 inoculated broilers) together with the data from experiments of the same design published earlier in van Bunnik et al. ^2^ (included to increase the sample size). The estimates for parameters were as follows: the decay rate parameter α=0.000 day^-1^ (CI: 0- 0.083), the transmission rate parameter β=0.008 day^-1^ (CI: 0.005- 0.027), and the diffusion coefficient D=0.089 m^2^day^-1^ (CI: 0.026- 0.826). Univariate profile likelihoods are shown in Fig S1.


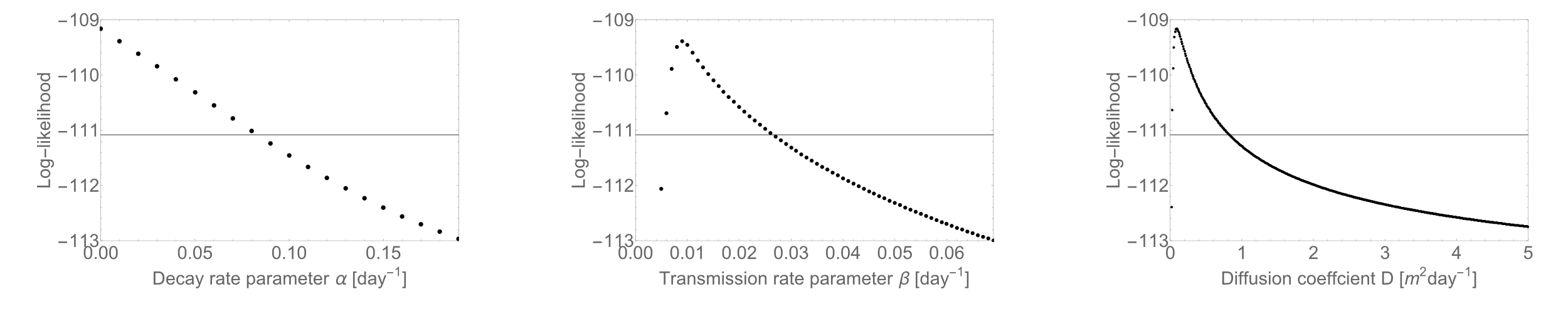


Fig S1. Profile likelihoods for model parameters estimated with previously published data on a single distance band only: decay rate parameter α, transmission rate parameter β and diffusion coefficient D. Horizontal lines mark the likelihood value for the confidence bounds.

Supplementary Note 3: Model fit and validation for fixed decay rate α=2.25 day^-1^

The decay rate value α=2.25 day^-1^ was estimated in a separate *C. jejuni* survival experiment, where faeces stored in the same environmental conditions as those used in the transmission experiments were sampled and Campylobacter enumeration was performed, as mentioned in Supplementary Note 2 and explained in van Bunnik et al. ^1^. The decay rate estimate was obtained by fitting an exponential curve to the temporal data. See van Bunnik et al. ^1^ for details.

For the model fitting here, we set α=2.25 day^-1^ and estimated the two remaining parameters from all the data from our transmission experiments. Here, the same approach for likelihood formulation and parameter estimation, namely automatic likelihood formulation and a three-step maximization procedure, was used as presented in the main manuscript for the estimation of all three model parameters (for details see ‘*Likelihood formulation and parameter estimation’* paragraph in the Methods section), except that confidence bounds were calculated with lower accuracy.

The likelihood was maximized for two remaining parameters: the transmission rate parameter was estimated to be β=4.32 day^-1^ [CI: 2.2-7.8] and the diffusion coefficient was estimated to be D=0.15 m^2^day^-1^ [CI: 0.10-0.23]. Univariate profile likelihoods for both parameters are shown in Fig S2.


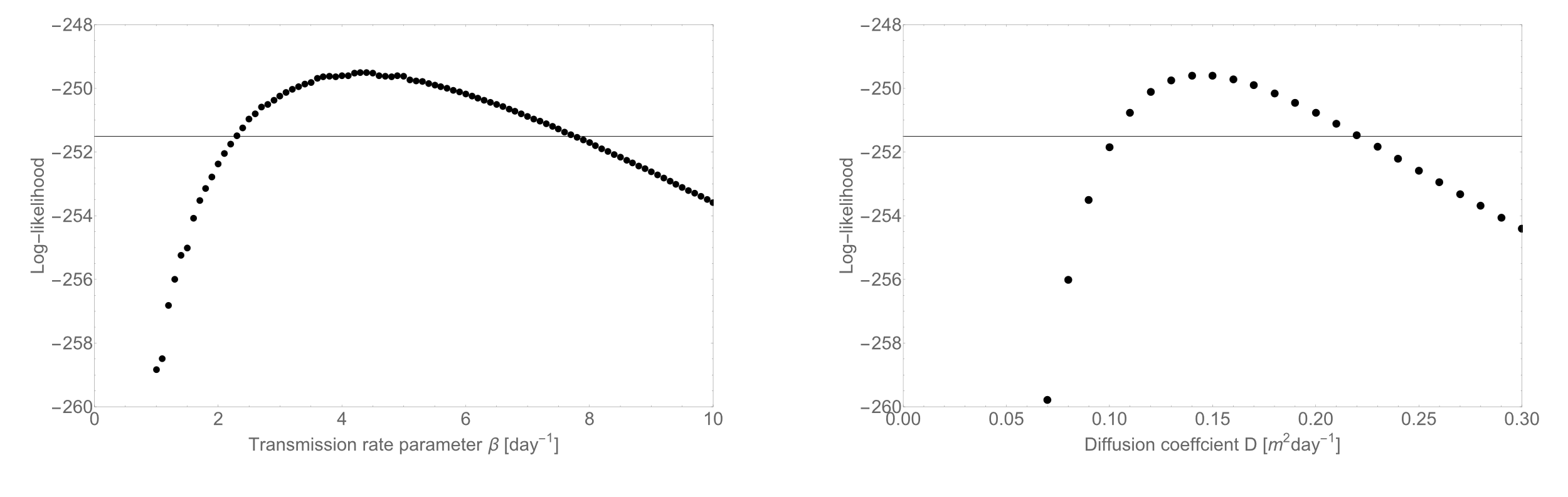

Fig S2. Profile likelihoods for model parameters for a model with fixed decay rate α=2.25 day^-1^: transmission rate parameter β and diffusion coefficient D; horizontal lines mark the likelihood value for the confidence bounds.

The Akaike Information Criterion (AIC) value was calculated from maximum loglikelihood values for this model with fixed α=2.25 day^-1^ (2 parameters; AIC: 503.169) and similarly for the original model where all three parameters were estimated together (3 parameters; AIC: 490.125). The difference in AIC is bigger than 10 which indicates that the original model, where all three parameters were estimated, provides a significantly better fit ^3^.

To assess the fit, the same methodology was used as presented in the main text for the model with estimation of all three model parameters; the model fits and experimental data were aggregated into spatiotemporal bins and the probability mass function (PMF) for number of colonised pens in each spatiotemporal bin was calculated for model fit parameters using the Poisson Binomial distribution and from the PMFs the p-value was calculated as the probability of observing the particular experimental outcome or more extreme values (for details see ‘*Statistical analysis of model fit’* paragraph in the Methods section of the main manuscript) The results for the type 1 experiments are presented in Fig S3, the results for the type 2 experiments are presented on Fig S4 (group A) and Fig S5 (group B & C).


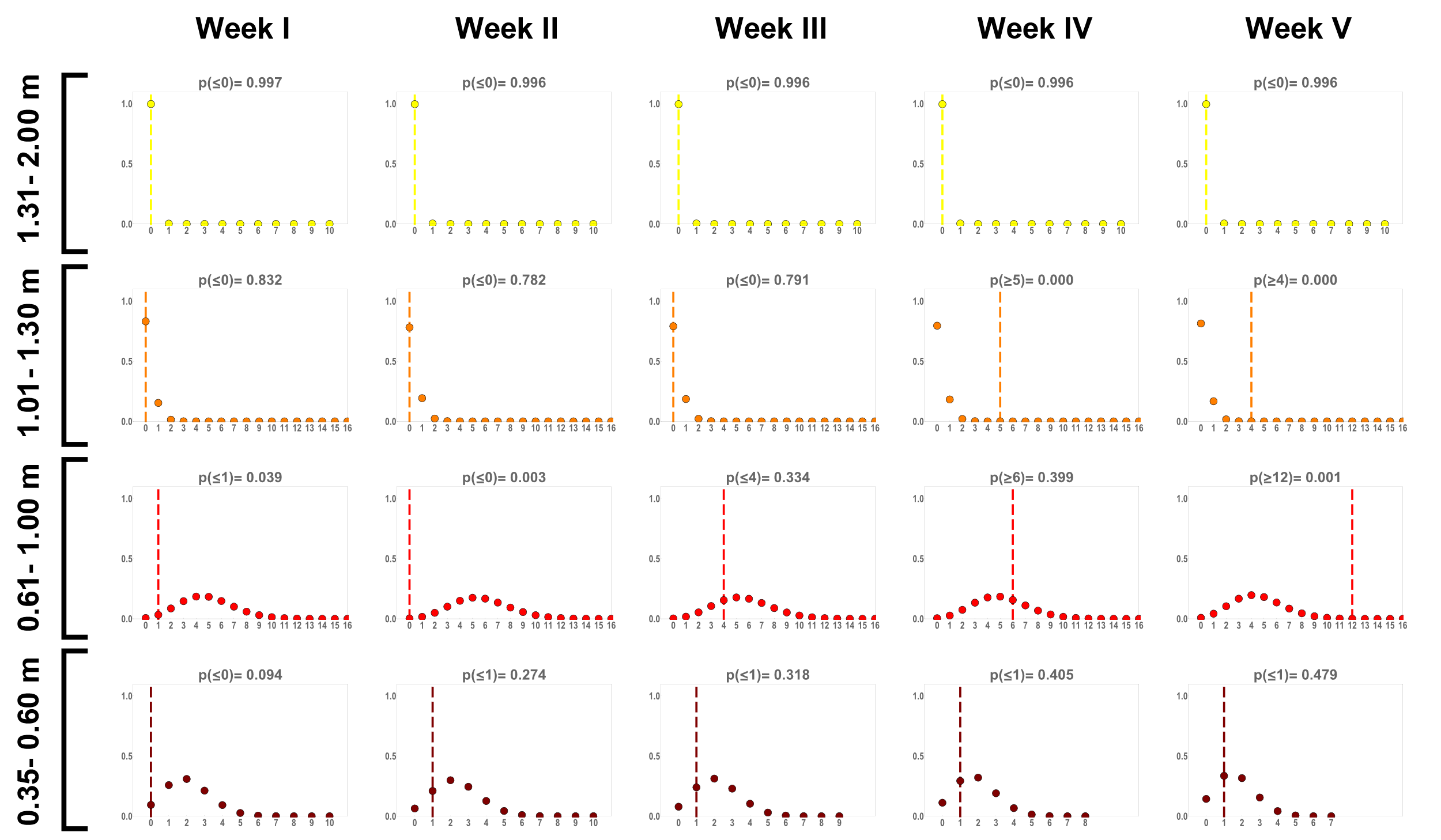


Fig S3. Probability mass functions generated from model predictions for model with fixed α=2.25 day^-1^ for type 1 experiments representing total number of cases per 1 spatiotemporal bin of 1 week for pens grouped into four distance bins: 0.35-0.60 m, 0.61-1.00 m, 1.01-1.30 m, 1.31-2.00 m; on the x axis is the number of positive cases observed during a 1-week interval, and the y axis shows the probability. The vertical line marks the total number of cases observed for the particular bin.

**
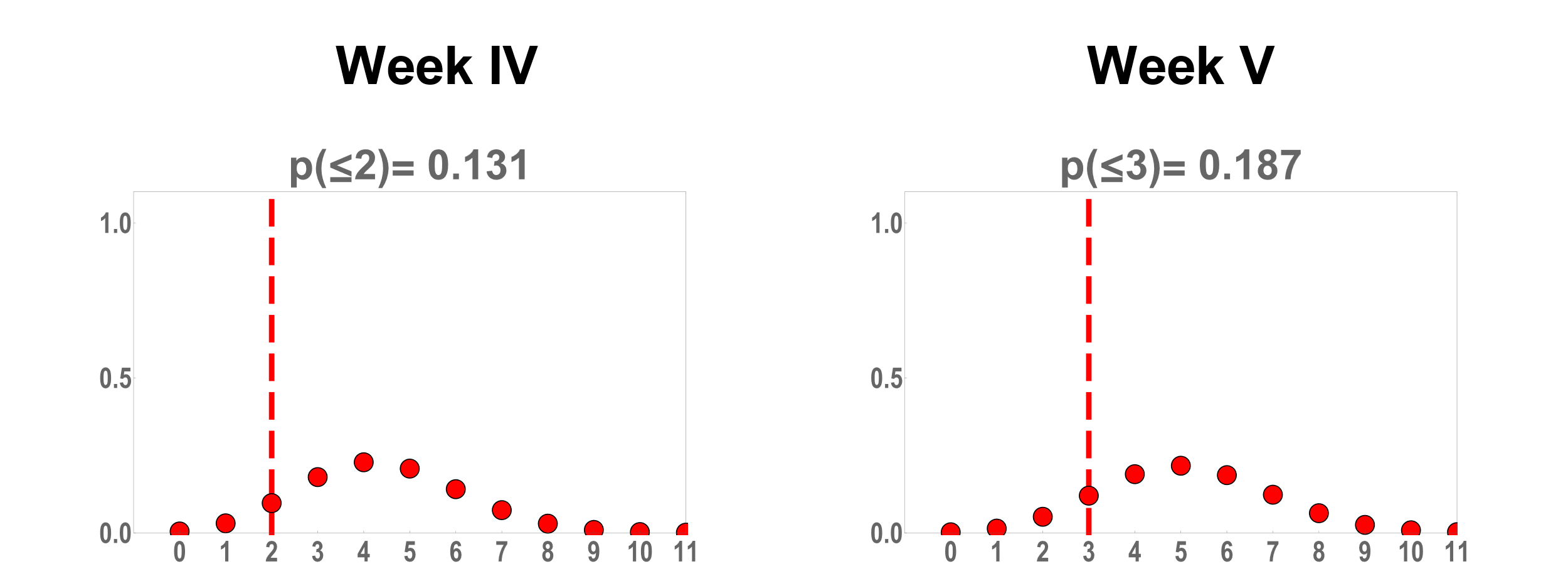
**

Fig S4. Probability mass functions generated from model predictions with fixed α=2.25 day^-1^ in type 2 experiment for group A representing total number of cases per week; on the x axis is the number of positive cases observed during a 1-week interval, and the y axis shows the probability. The vertical line marks the outcome observed in the experiments.

**
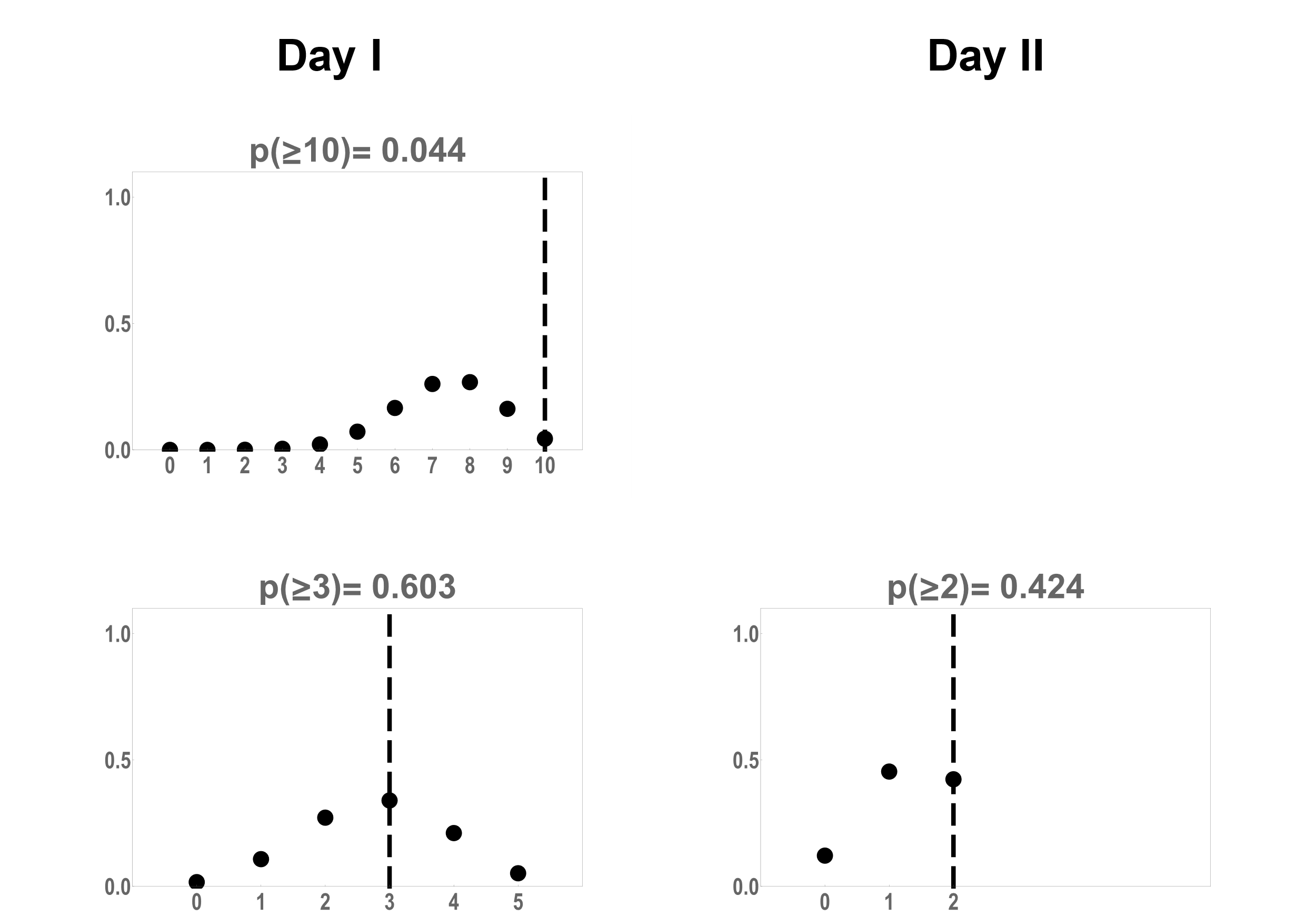
**

Fig S5. Probability mass functions generated from model predictions with fixed α=2.25 day^-1^ in type 2 experiment for group B & C (pairwise groups) representing total number of cases per day; on each plot the x axis is the number of positive cases observed during a 1-day interval, and the y axis shows the probability. The vertical line marks the outcome observed.

P-value for the Fisher's combined probability test for p-values of all the spatiotemporal bins form all the experiments was: 0.000637. P-values for all spatiotemporal bins and combinations of thereof are presented in Table S3 and Table S4.

Table S3. P-values for all spatiotemporal bins for type 1 experiments with p values for the Fisher's combined probability test (FCP) for model with fixed α=2.25 day^-1^

|  | Week 1 | Week 2 | Week 3 | Week 4 | Week 5 | FCP |
| --- | --- | --- | --- | --- | --- | --- |
| Bin 4 | 0.997422 | 0.996178 | 0.996177 | 0.996178 | 0.996178 | **1.000000** |
| Bin 3 | 0.831535 | 0.781862 | 0.791078 | 0.000003 | 0.000052 | **0.000001** |
| Bin 2 | 0.039308 | 0.002585 | 0.334424 | 0.398640 | 0.001385 | **0.000099** |
| Bin 1 | 0.094025 | 0.274093 | 0.318458 | 0.405202 | 0.479225 | **0.230265** |
| FCP | **0.171188** | **0.059063** | **0.762311** | **0.000329** | **0.000035** | **0.000002** |

Table S4. P-values for all spatiotemporal bins for type 2 experiment with p-values for the Fisher's combined probability test (FCP) for model with fixed α=2.25 day^-1^

|  | Week 4 | Week 5 | FCP |
| --- | --- | --- | --- |
| Group A | 0.130534 | 0.186576 | **0.114832** |
|  | Day 1 | Day 2 | **FCP** |
| Group B | 0.044103 |  | **0.044103** |
| Group C | 0.603160 | 0.423824 | **0.604320** |
| FCP | **0.123053** | **0.423824** | **0.175216** |

Supplementary Note 4: Additional analysis

1. Experiment type 2 group C- contributions of main and additional sources

For the group C from the type 2 experiment (pairwise transmission in recipient pens) we used the fitted model with point estimate values for all three parameters α=0.153 day^-1^, D=0.013 m^2^day^-1^ and β=0.372 day^-1^ and evaluated the contribution to the probability of infection from the main source (which is the pen mate) to the recipient as well as the contribution from all the other sources (central area source pen and other recipient pens if they became infected before).

Table S5. Mean probability of infection estimated from our model (with point estimate values of parameters α=0.153 day^-1^ , D=0.013 m^2^day^-1^ and β=0.372 day^-1^) for pens in type 2 experiment: group B (n=10 pens) and group C (n=5 pens); additionally for group C the contribution to probability was estimated separately for main source (their pen mate) and additional sources (separated by distance).

|  | | Day 1^1^ | Day 2^1^ | Day 3^1^ |
| --- | --- | --- | --- | --- |
| Group B | from all sources together | 0.783854 | 0.816726 | 0.859933 |
| Group C | from all sources together | 0.179582 | 0.414533 | 0.591947 |
|  | from main source only | 0.138976 | 0.317660 | 0.423870 |
|  | from additional sources only | 0.039521 | 0.041396 | 0.043592 |

1. Day of exposure to the main source
2. Removed (potential) sources contribution for type 1 experiment

For type 1 experiments recipient broilers that were tested positive were removed immediately once cloacal swab collected from them was detected positive for *C. Jejuni*; in modelling, those broilers were not included as new sources because they were shedding only for a short period.

To estimate the effect that these additional sources may have on transmission we use point estimate values of the model parameters α=0.153 day^-1^, D=0.013 m^2^day^-1^ and β=0.372 day^-1^ to calculate what contribution these sources would make to the probability of transmission.


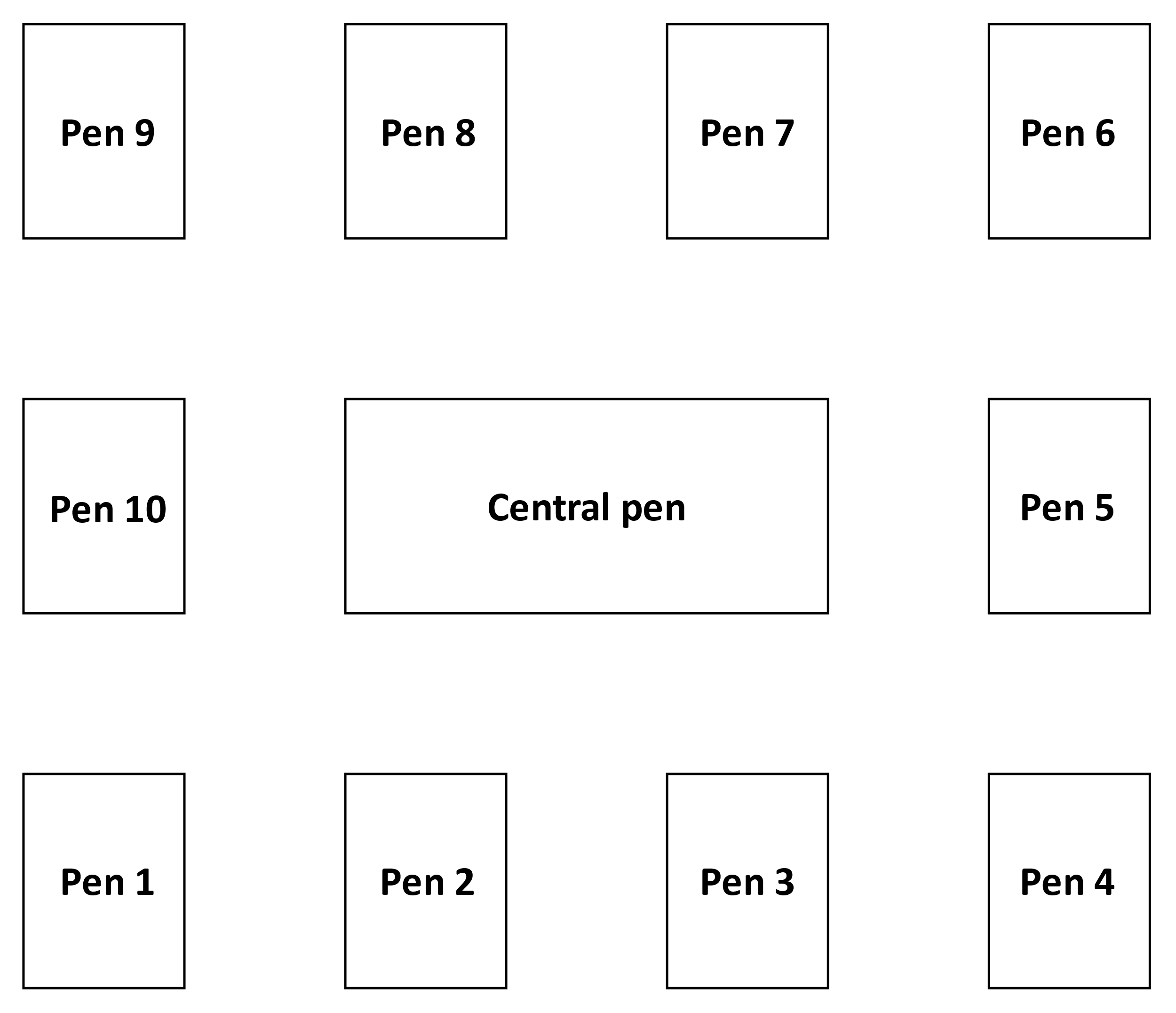


Fig S6. Schematic representation of experimental room with named pens from type 1 experiments 1-3.

As an example we consider one of the pens (pen 2, see Fig S6) separated from the central pen and neighboring pens by 0.75 m (border to border distance) as a source and calculated its contribution to the probability of infection in the remaining 9 pens and compared it to the contribution from the central pen (main source); the results are presented in Table S6 and Table S7.

Table S6. Probability of being infected by 1 source broiler housed in pen 2 that started shedding on day 1 for all remaining pens for 5 consecutive days.

|  | Day 1 | Day 2 | Day 3 | Day 4 | Day 5 |
| --- | --- | --- | --- | --- | --- |
| Pen 1 | 1.50∙10^-10^ | 5.44∙10^-07^ | 1.64∙10^-05^ | 1.06∙10^-4^ | 3.46∙10^-4^ |
| Pen 3 | 1.50∙10^-10^ | 5.44∙10^-07^ | 1.64∙10^-05^ | 1.06∙10^-4^ | 3.46∙10^-4^ |
| Pen 4 | 0 | 0 | 0 | 4.44∙10^-16^ | 1.44∙10^-13^ |
| Pen 5 | 0 | 0 | 0 | 0 | 4.44∙10^-16^ |
| Pen 6 | 0 | 0 | 0 | 0 | 0 |
| Pen 7 | 0 | 0 | 0 | 0 | 0 |
| Pen 8 | 0 | 0 | 0 | 0 | 6.66∙10^-16^ |
| Pen 9 | 0 | 0 | 0 | 0 | 0 |
| Pen 10 | 0 | 7.51∙10^-12^ | 2.42∙10^-09^ | 6.31∙10^-08^ | 5.15∙10^-07^ |

Table S7. Probability of being infected by 5 source broilers housed in the central pen that started shedding on day 1 for all remaining pens for 5 consecutive days.

|  | Day 1 | Day 2 | Day 3 | Day 4 | Day 5 |
| --- | --- | --- | --- | --- | --- |
| Pen 1 | 0 | 1.25∙10^-11^ | 4.03∙10^-09^ | 1.05∙10^-07^ | 8.58∙10^-07^ |
| Pen 3 | 1.96∙10^-10^ | 7.26∙10^-07^ | 2.21∙10^-05^ | 1.46∙10^-04^ | 4.82∙10^-04^ |
| Pen 4 | 0 | 1.25∙10^-11^ | 4.03∙10^-09^ | 1.05∙10^-07^ | 8.58∙10^-07^ |
| Pen 5 | 2.50∙10^-10^ | 9.07∙10^-07^ | 2.73∙10^-05^ | 1.77∙10^-04^ | 5.76∙10^-04^ |
| Pen 6 | 0 | 1.25∙10^-11^ | 4.03∙10^-09^ | 1.05∙10^-07^ | 8.58∙10^-07^ |
| Pen 7 | 1.96∙10^-10^ | 7.26∙10^-07^ | 2.21∙10^-05^ | 1.46∙10^-04^ | 4.82∙10^-04^ |
| Pen 8 | 1.96∙10^-10^ | 7.26∙10^-07^ | 2.21∙10^-05^ | 1.46∙10^-04^ | 4.82∙10^-04^ |
| Pen 9 | 0 | 1.25∙10^-11^ | 4.03∙10^-09^ | 1.05∙10^-07^ | 8.58∙10^-07^ |
| Pen 10 | 2.50∙10^-10^ | 9.07∙10^-07^ | 2.73∙10^-05^ | 1.77∙10^-04^ | 5.76∙10^-04^ |

1. Boundary conditions

To keep our model simple and easily computable, we assumed absorbing boundary conditions, treating each experimental room as an area of infinite size. In experiments, some pens in a room were placed near the wall, so theoretically it is possible that portion of infectious material that reached the wall stayed in the pen (instead of diffusing further) and influenced probability of infection. To assess how the boundary conditions assumption influenced our results, we compared the probability of infection calculated for point estimate parameter values (α=0.153 day^-1^, D=0.013 m^2^day^-1^ and β=0.372 day^-1^) for one, chosen recipient area from type 1 experiments 1-3 (pen 2, see Fig S6) to that for a recipient area that is extended (virtually) in the direction of the room wall by an area of the same size (virtual mirror pen).

For both areas the probabilities estimated for day 1, 10, 20 and 30 when exposed to the main source area (central pen) are shown in Table S8. The difference between these probability estimates is small, which supports our decision to use absorbing boundaries.

Table S8. Probability of being infected by 5 source broilers housed in the central pen that started shedding on day 1 for one chosen recipient area (pen 2) from type 1 experiment 1-3 and for area of doubled size extended in the direction of the room wall.

|  | Day 1 | Day 10 | Day 20 | Day 30 |
| --- | --- | --- | --- | --- |
| Original recipient area (0.75 m2) | 1.96∙10^-10^ | 0.006105 | 0.021043 | 0.027634 |
| Extended recipient area (1.5 m2) | 1.96∙10^-10^ | 0.006109 | 0.021249 | 0.028208 |
| Difference | 0 | 4.19∙10^-06^ | 0.000206 | 0.000574 |

Supplementary figures S7 to S15: Spatial organization of experimental rooms


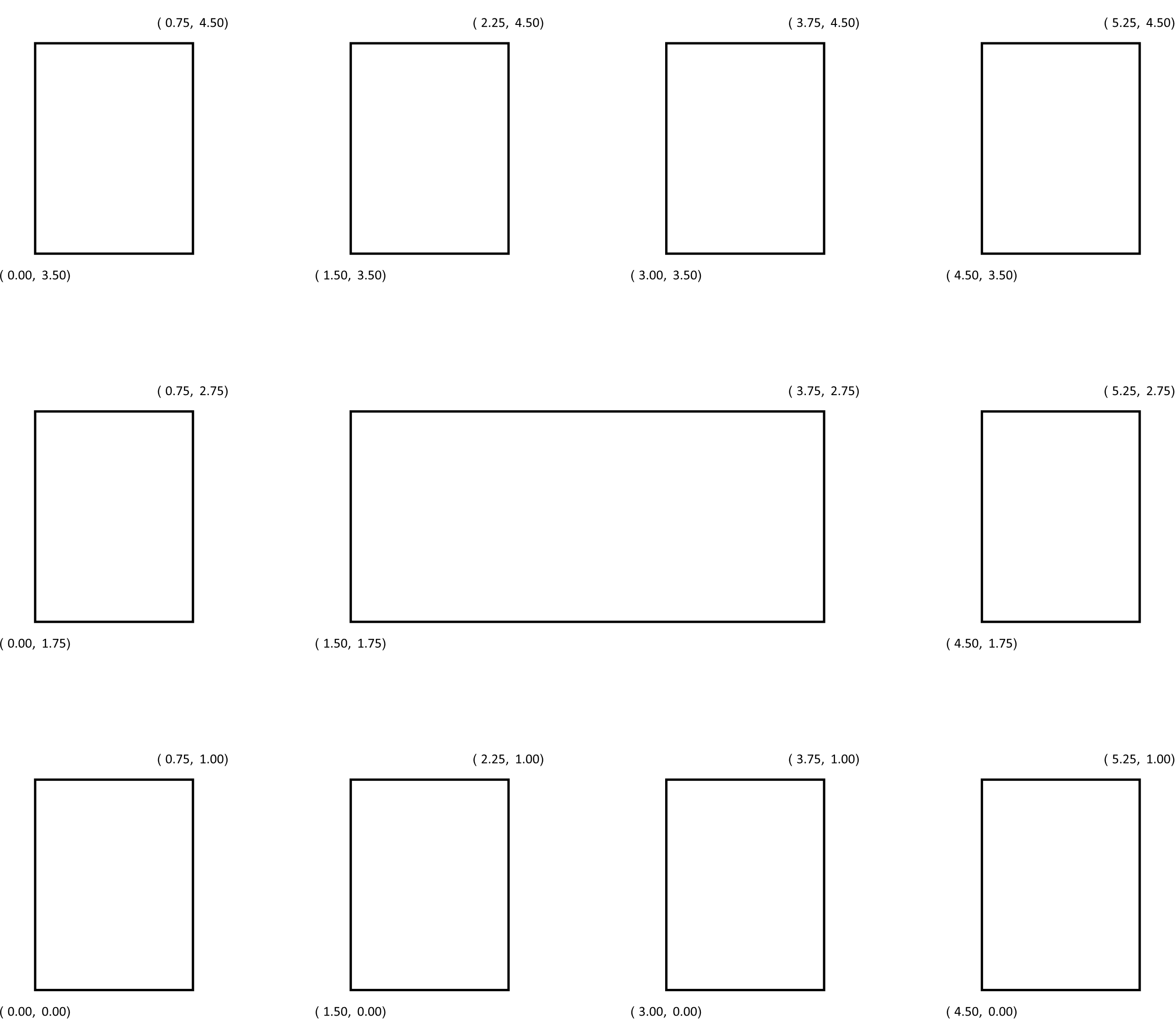

Fig S7. Schematic spatial organization for type 1 experiments 1-3, all rooms; as detailed between-pen distance measurements were not collected during these experiments, coordinates were prepared based on the assumption that all rooms had identical, symmetrical design.


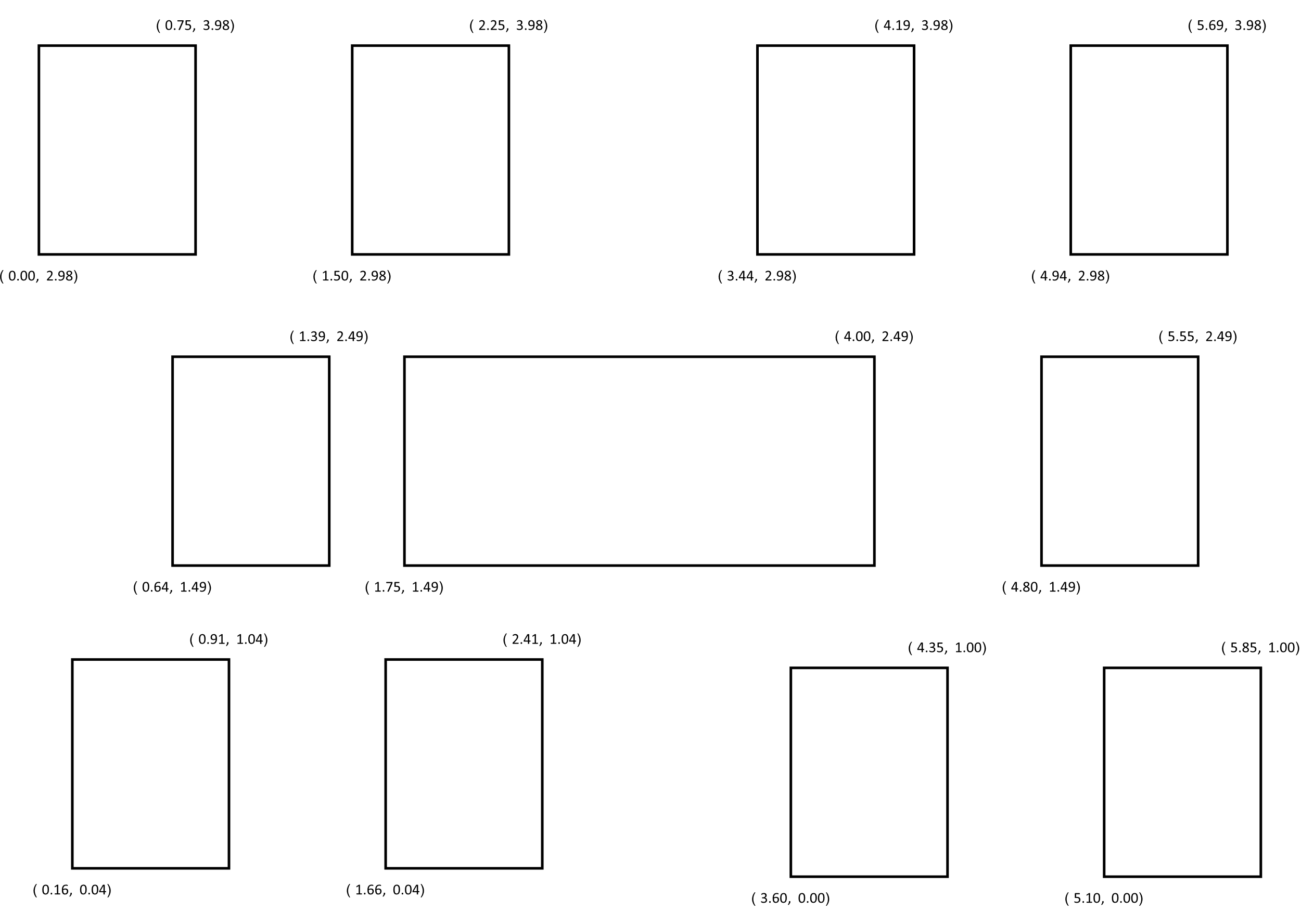

Fig S8. Spatial organization for type 1 experiment 4, room 1.


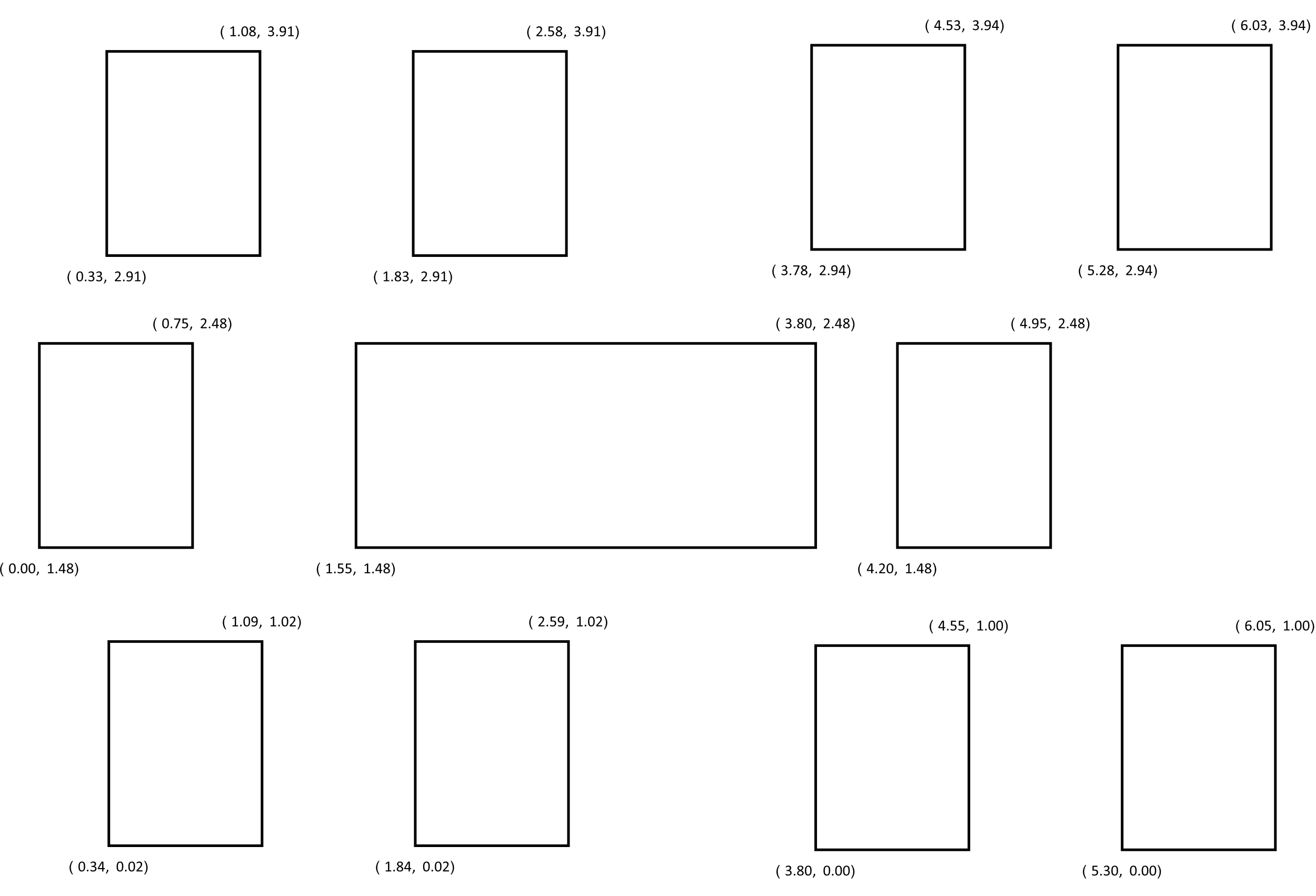

Fig S9. Spatial organization for type 1 experiment 4, room 2.


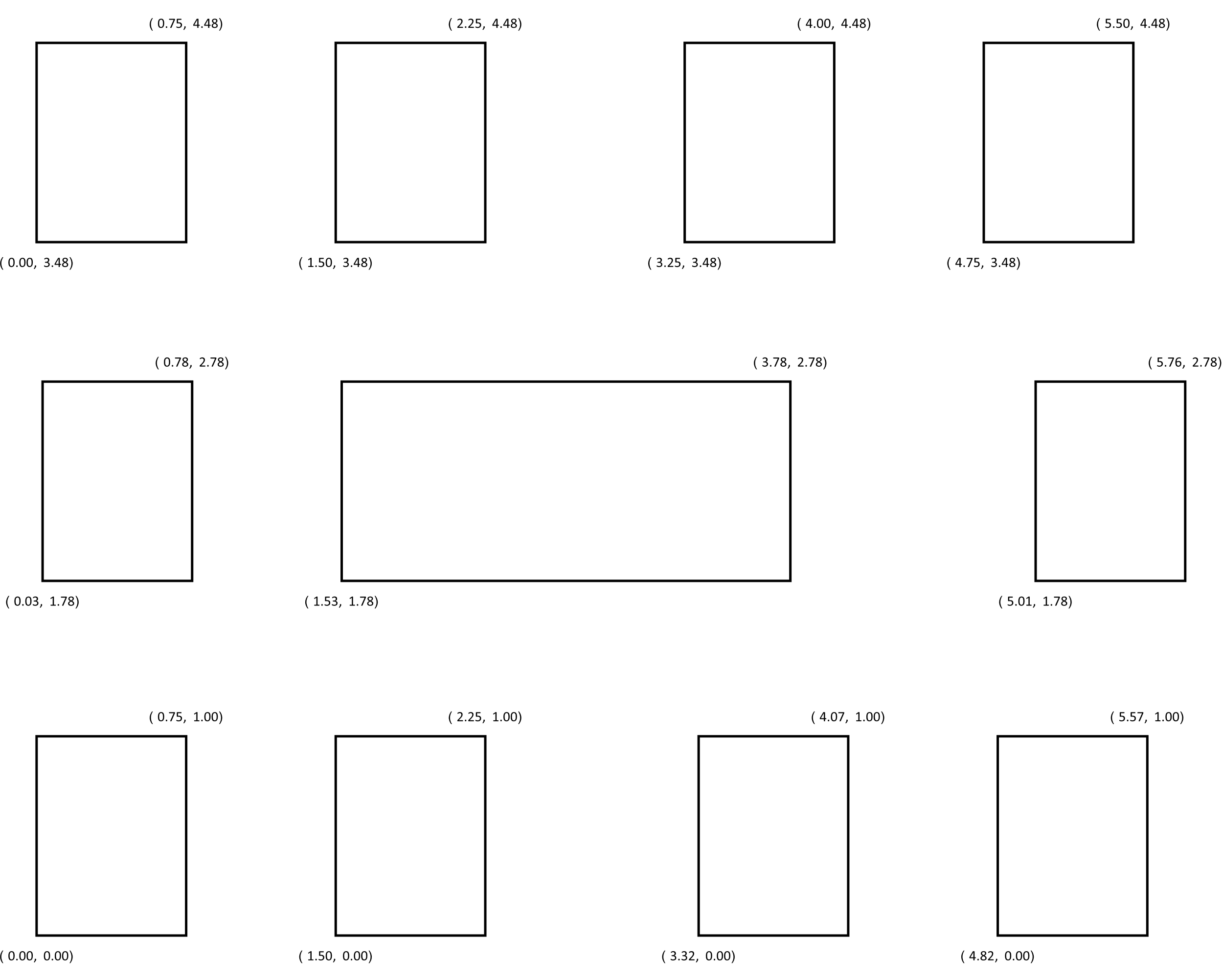

Fig S10. Spatial organization for type 1 experiment 4, room 3.


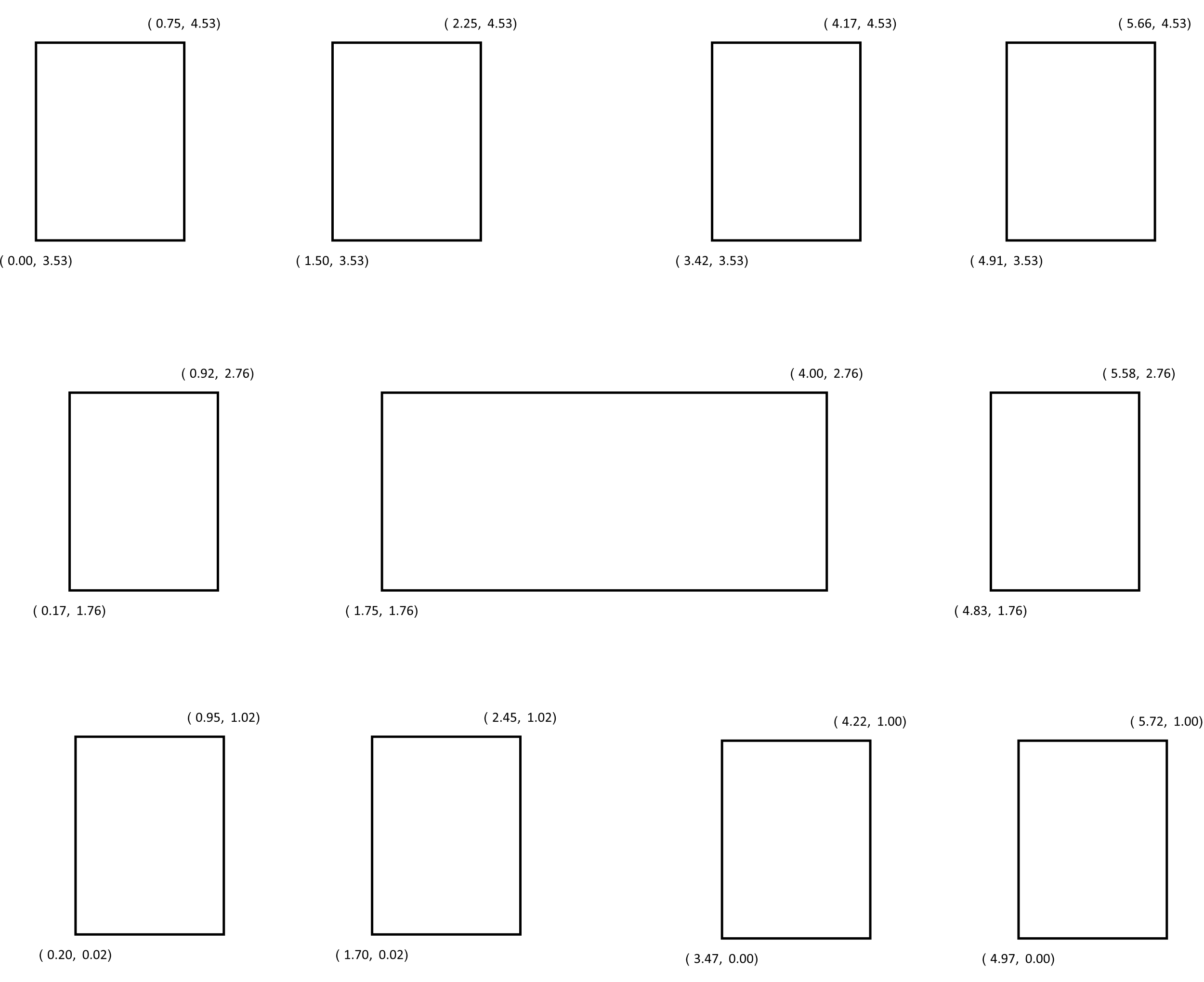

Fig S11. Spatial organization for type 1 experiment 4, room 4.


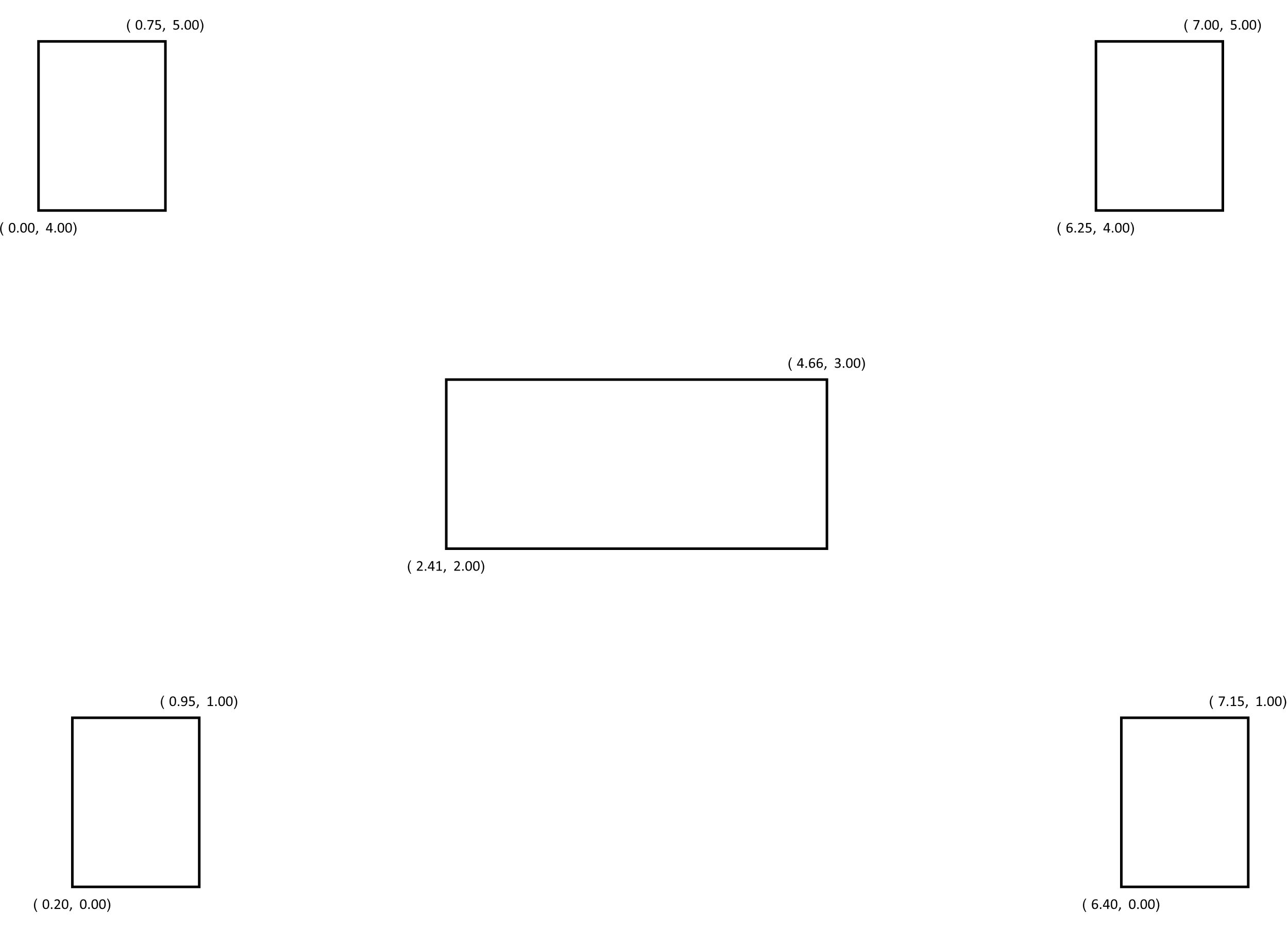


Fig S12. Spatial organization for type 1 experiments 4, room 5.


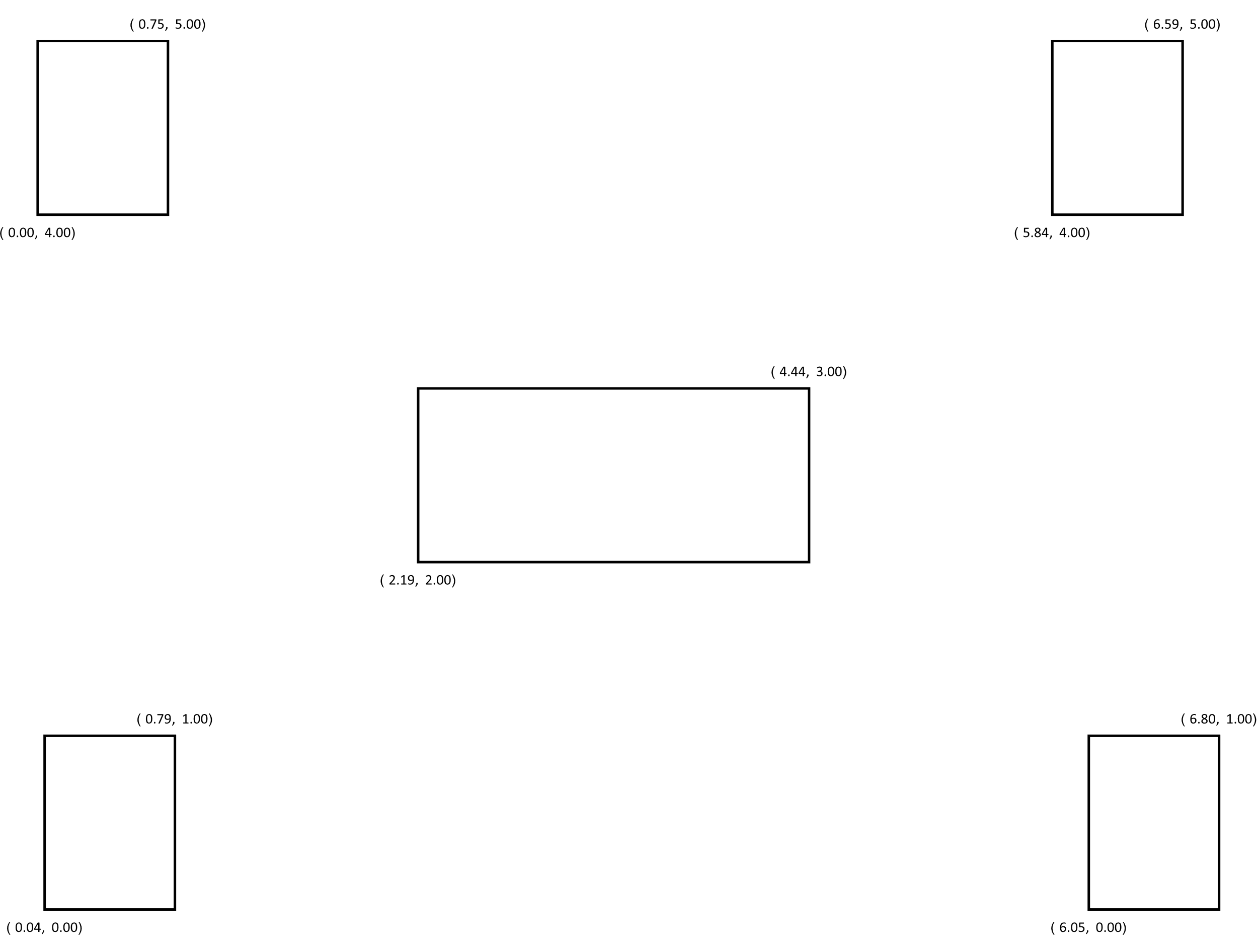

Fig S13. Spatial organization for type 1 experiment 4, room 6.


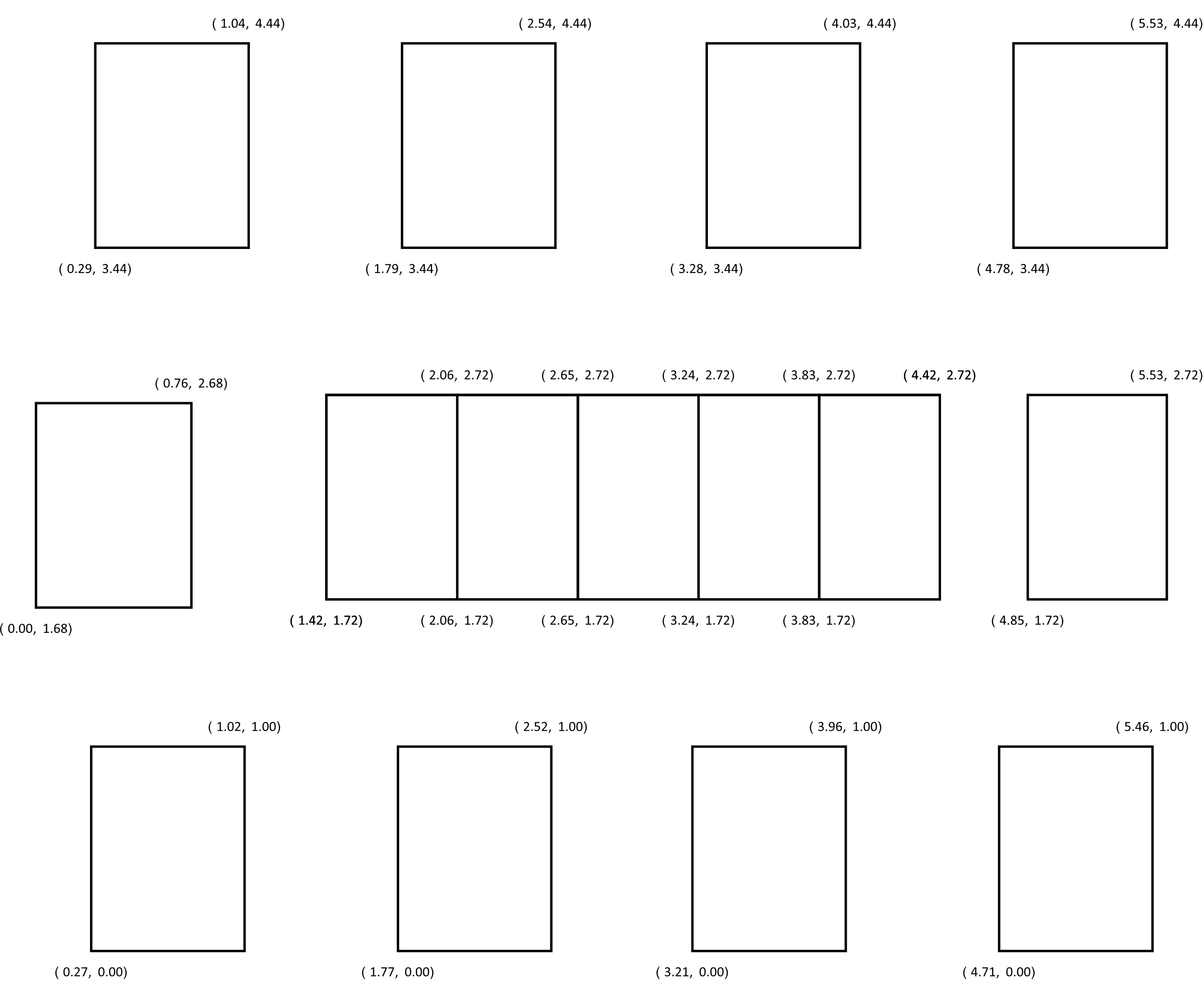

Fig S14. Spatial organization for type 2 experiment, room 1.


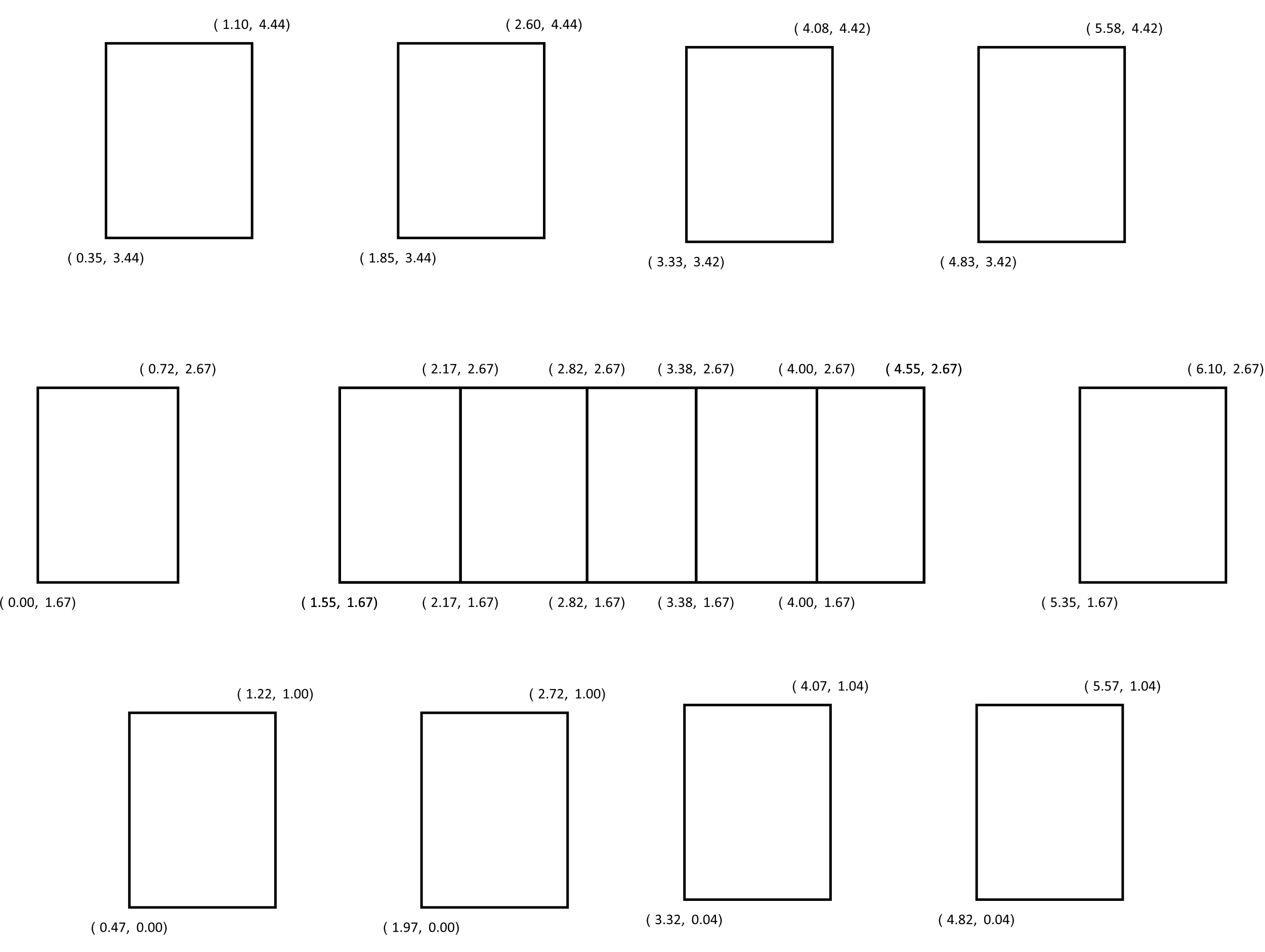

Fig S15. Spatial organization for type 2 experiment, room 2.

Supplementary References

1 van Bunnik, B. A. D. *et al.* Small distances can keep bacteria at bay for days. *Proceedings of the National Academy of Sciences* **111**, 3556-3560 (2014). <https://doi.org/10.1073/pnas.1310043111>

2 van Bunnik, B. A. D., Hagenaars, T. J., Bolder, N. M., Nodelijk, G. & de Jong, M. C. M. Interaction effects between sender and receiver processes in indirect transmission of Campylobacter jejuni between broilers. *BMC Veterinary Research* **8**, 123 (2012). <https://doi.org/10.1186/1746-6148-8-123>

3 in *Model Selection and Multimodel Inference: A Practical Information-Theoretic Approach* (eds Kenneth P. Burnham & David R. Anderson) 149-205 (Springer New York, 2002).
